# Increased probability of hot and dry weather extremes during the growing season threatens global crop yields

**DOI:** 10.1038/s41598-023-29378-2

**Published:** 2023-03-03

**Authors:** Matias Heino, Pekka Kinnunen, Weston Anderson, Deepak K. Ray, Michael J. Puma, Olli Varis, Stefan Siebert, Matti Kummu

**Affiliations:** 1 Water and Development Research Group, Aalto University, Finland, Tietotie 1E, 02150 Espoo, Finland; 2 International Research Institute for Climate and Society, Columbia University, Palisades, NY 10964 USA; 3 Earth System Science Interdisciplinary Center, University of Maryland, College Park, MD USA; 4 Institute On the Environment, University of Minnesota, Saint Paul, MN USA; 5 Center for Climate Systems Research, Columbia University, 2880 Broadway, New York, NY 10025 USA; 6 NASA Goddard Institute for Space Studies, 2880 Broadway, New York, NY 10025 USA; 7 Department of Crop Sciences, University of Goettingen, Von-Siebold-Str. 8, 37075 Goettingen, Germany

**Keywords:** Climate sciences, Environmental sciences

## Abstract

Although extreme weather events recur periodically everywhere, the impacts of their simultaneous occurrence on crop yields are globally unknown. In this study, we estimate the impacts of combined hot and dry extremes as well as cold and wet extremes on maize, rice, soybean, and wheat yields using gridded weather data and reported crop yield data at the global scale for 1980–2009. Our results show that co-occurring extremely hot and dry events have globally consistent negative effects on the yields of all inspected crop types. Extremely cold and wet conditions were observed to reduce crop yields globally too, although to a lesser extent and the impacts being more uncertain and inconsistent. Critically, we found that over the study period, the probability of co-occurring extreme hot and dry events during the growing season increased across all inspected crop types; wheat showing the largest, up to a six-fold, increase. Hence, our study highlights the potentially detrimental impacts that increasing climate variability can have on global food production.

Understanding the weather signal in crop yield variations is imperative for efforts to adapt agricultural production to climate change. Historically, approximately a third of global crop yield variability can be explained by weather^1^, with major impacts on food systems caused by severe extreme weather events^2^. The sensitivity of crop yields to weather varies strongly across regions, and some globally important crop production areas such as maize in Midwestern United States and rice in Japan tend to show a greater fraction of yield variations explained by weather^1,3–5^.

Typically, plants have a narrow climatic range corresponding to optimal growing conditions; outside of this range, their growth is decreased^6–10^ or even collapsed^11^. Previous research has shown that excessive heat^2,6,10,12^ and drought^13,14^ consistently reduce crop yields, while the impacts of anomalously wet^15,16^ and cold^13^ weather have been more diverse. However, in regional studies, wet weather has been observed to impose major yield reductions, comparable to those of droughts^17,18^. Worryingly, the frequency of extreme weather occurrences has increased in recent decades^19–22^, and the trend is projected to continue in the future^23,24^. Hence, understanding the historical trends in the co-occurrence of extreme weather events, and their related impacts on agriculture should be a global priority.

Most of the studies described above have presented the impacts of individually occurring extreme weather events^2,7,13^. To our knowledge, previous global studies that have investigated the effects of co-occurring extremes were limited to single crop types or have utilized national level data^15,25,26^. Here, we address this gap by utilizing sub-nationally reported and gridded global data on crop yields^27^ to investigate how combined (in the same place at the same time) hot-dry and cold-wet extreme events have impacted wheat, maize, soybean, and rice yields at the global scale. Further, to understand whether hazards related to these co-occurring extremes have historically changed, we quantified how their probability during the growing season has developed in recent years (1980–2009).

For analysing the dependencies between climate variability and crop yield anomalies, we used growing season weather indicators calculated from daily time series of temperature^28^, soil moisture^29^, and precipitation^28^ over 1980–2009 at 0.5º resolution together with annual wheat, maize, soybean, and rice yield data reported for approximately 20,000 political units and disaggregated to grid scale^27^. Namely, we developed boosted regression tree models^30^ with the XGBoost algorithm^31^ to quantify the relationship between co-occurring hot and dry (cold and wet) conditions and crop yield anomalies. Finally, we assessed whether the probability of these events has changed in recent history.

## Results

### Large differences in sensitivity of crop yield to climate variability across crops and regions

To estimate the sensitivity of annual crop yield anomalies to climate variations, we developed a machine learning based model using the XGBoost algorithms for reported crop yields and growing season weather data at the global and climate bin scale (Table 1). We first assessed the performance of the model by calculating how much of the interannual variation of crop yield anomalies the model was able to explain and compared those numbers to existing studies. For each spatial unit, the train-test split was conducted so that crop yield anomalies were always estimated for years that were not used in model training, which means that they out-of-sample predictions (see “Methods”, Fig. S1).

**Table 1:** The variables of the main analyses (Figs. 1, 2, and 3) and their sources. The variables were calculated for each crop, year, and grid cell. For hot, and dry (cold, and wet) days, we calculated the days above (below) 90th (10th) percentiles for temperature and soil moisture deficit across all years. All indicators were calculated for the growing season except one precipitation indicator, which was calculated for the whole year proceeding harvest. See “Methods” for a more elaborate description about the data processing as well as Table S1 for a comprehensive list of the modelling set-ups conducted in this study.

Variable	Source
Hot days	AgMerra^28^
Dry days	ERA5^29^
Wet days	ERA5
Cold days	AgMerra
Average temperature	AgMerra
Average soil moisture	ERA5
Total precipitation	AgMerra
Total precipitation (whole year)	AgMerra
Crop yield anomaly	Ray et al. (2019)^27^

**Figure 1:**
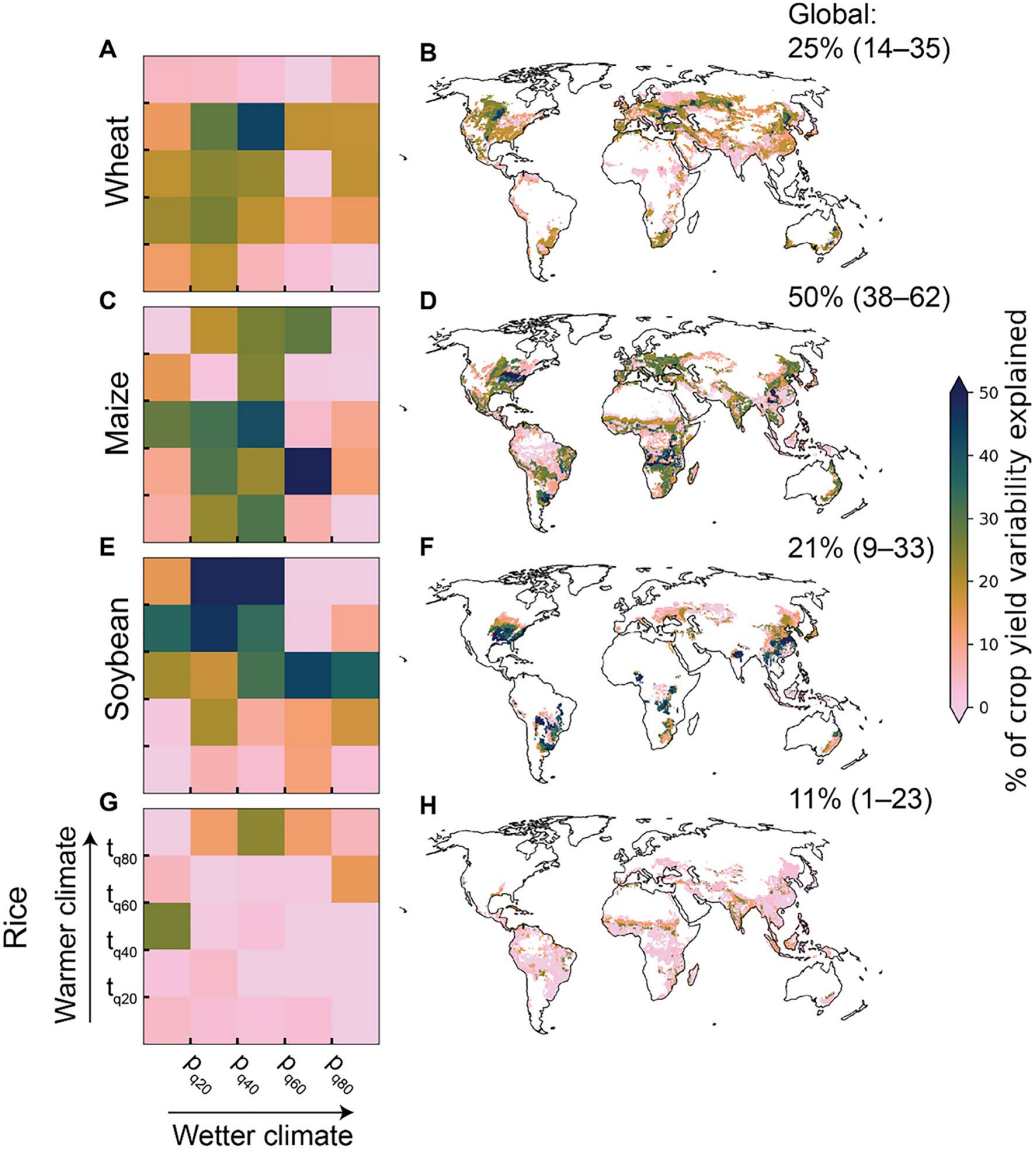
Explanatory power of the XGBoost regression model in estimating crop yield anomalies with growing season weather data at global and climate bin level. Results are shown for studied crops: wheat (**A**,**B**), maize (**C**,**D**), soybean (**E**,**F**), and rice (**G**,**H**). See map and division of the climate bins, defined based on quintiles of climatological temperature (t) and precipitation (p), for each crop in Fig. S2. The XGBoost model was trained globally as well as for each climate bin separately (see “Methods”). The results presented in the climate bin matrices and maps show the amount of crop yield variability explained with the models trained for each climate bin separately, whereas the global results are presented in the upper right corner of each map. The crop yield anomalies were estimated for each grid cell separately and always for years, which were not used in training the model (i.e. the estimates are out-of-sample predictions). Uncertainty in the global results was evaluated by training the model 100 times, while randomly sampling the years in each training and validation set. The proportion of crop yield variation explained by the models was calculated as the squared Pearson’s correlation coefficient (sign preserved) between reported and estimated crop yield anomalies aggregated (with a harvested area-weighted average) to global and climate bin level. For the global results, the values in the brackets indicate 95% confidence interval of the proportion of crop yield anomaly variability explained by the models. The maps presented in the figure were created with Matplotlib and Cartopy Python modules^38–40^.

The model was able to explain 25% (95% confidence interval: 14–35%), 50% (38–62%), 21% (9–33%) and 11% (1–23%) of interannual variations in global wheat, maize, soybean, and rice yield anomalies, respectively (Fig. 1). The results are comparable to previous estimates based on both statistical^2^ and process-based modelling^4^, and they remain relatively similar with varying growing season set-up, soil moisture data, and choice of machine learning algorithm as well as irrespective whether average climatic conditions were considered (Table S1, Figs S7–S11). However, the proportion of explained crop yield anomaly variability is smaller when utilizing solely extreme climate indicators or a different crop yield data set for training and evaluating the models (Table S1, Figs. S12–S13).

We found large differences in the susceptibility of crop yield to weather with models trained separately for each climate bin (Fig. 1). The climate bins used here divide the crop-specific growing areas into 25 climatologically analogous regions with approximately equal sample size, based on temperature and precipitation (Fig. S2, see “Methods”). Often, weather explained less of the crop yield anomaly variability in climate bins located close to the equator, compared to those in higher latitudes (Fig. 1). This could potentially be due to, for example, generally highly suitable conditions for plant growth^32^, and lower data quality (e.g. because of smaller resources for weather monitoring and collection of agricultural statistics)^1^. Also differences in baseline yield variability (Fig. S5) due to, e.g., irrigation^33^ can affect the amount of crop yield variability explained by weather (Fig S3).

Our results (Fig. 1B) estimate that approximately 25% of global interannual wheat yield anomaly variability can be explained by weather which is comparable against results obtained from the average gridded global crop model^4^. However, the relationship was weaker compared to recent statistical global (46%)^2^ and regional (43%)^34^ estimates, potentially because we include a larger proportion of global wheat production in this study. Based on the climate bin specific models, wheat yields were most sensitive to weather variations in climate bins located in western parts of North America, eastern China, and Europe as well as southern South America, Australia, and Africa. (Fig. 1A,B). Interestingly, the susceptibility of wheat yield to weather was smaller in climate bins with higher irrigation use (e.g., in northern India and Pakistan) (Fig. S3A).

Out of the crop types studied, global maize yield showed largest susceptibility to climate variations, with 38–62% of crop yield anomaly variability explained by the models (Fig. 1), which aligns with previous results based on both statistical and process-based modelling^1,2,4^. For maize, the largest explanatory power was found in relatively cool and dry climate bins located, for example, in North America and China (Fig. 1D), both of which are globally important areas for maize production. Generally, those climate bins, where climate variability has the strongest relationship with maize yield anomalies tend to also have larger production (Fig. S4B).

For soybean, our analyses show similar extent of yield anomaly variability explained by weather compared to previous global estimates (2, 4). When training models across climate bins, strongest explanatory power was observed in relatively warm and dry regions, which include for example parts of North, and South America, China, and India (Fig. 1E,F). These are all areas, where climate variations have also previously been reported to explain the variability of soybean yield anomalies to a relatively large extent (~ 26% to ~ 46%)^1^.

Out of the crops studied here, rice yields showed the lowest susceptibility to climate variability (Fig. 1G,H). This has also been observed by other studies, which report rice yield anomalies to be less sensitive to climate variability at the global scale compared to other crop types^4^ and have shown that climate variations cannot explain rice yield variability in approximately 48% of global rice harvested areas^1^. The difficulty with explaining global rice yield variability with weather might be due to larger proportions of rice being irrigated compared to other crop types (Fig. S3)^35^, grown in warm regions that are water abundant (i.e. the tropics)^32^, and extensive practice of multiple cropping^36^ as well as a huge amount of existing varieties^37^.

### Co-occurring heat and drought have the largest yield-reducing impacts

**Figure 2:**
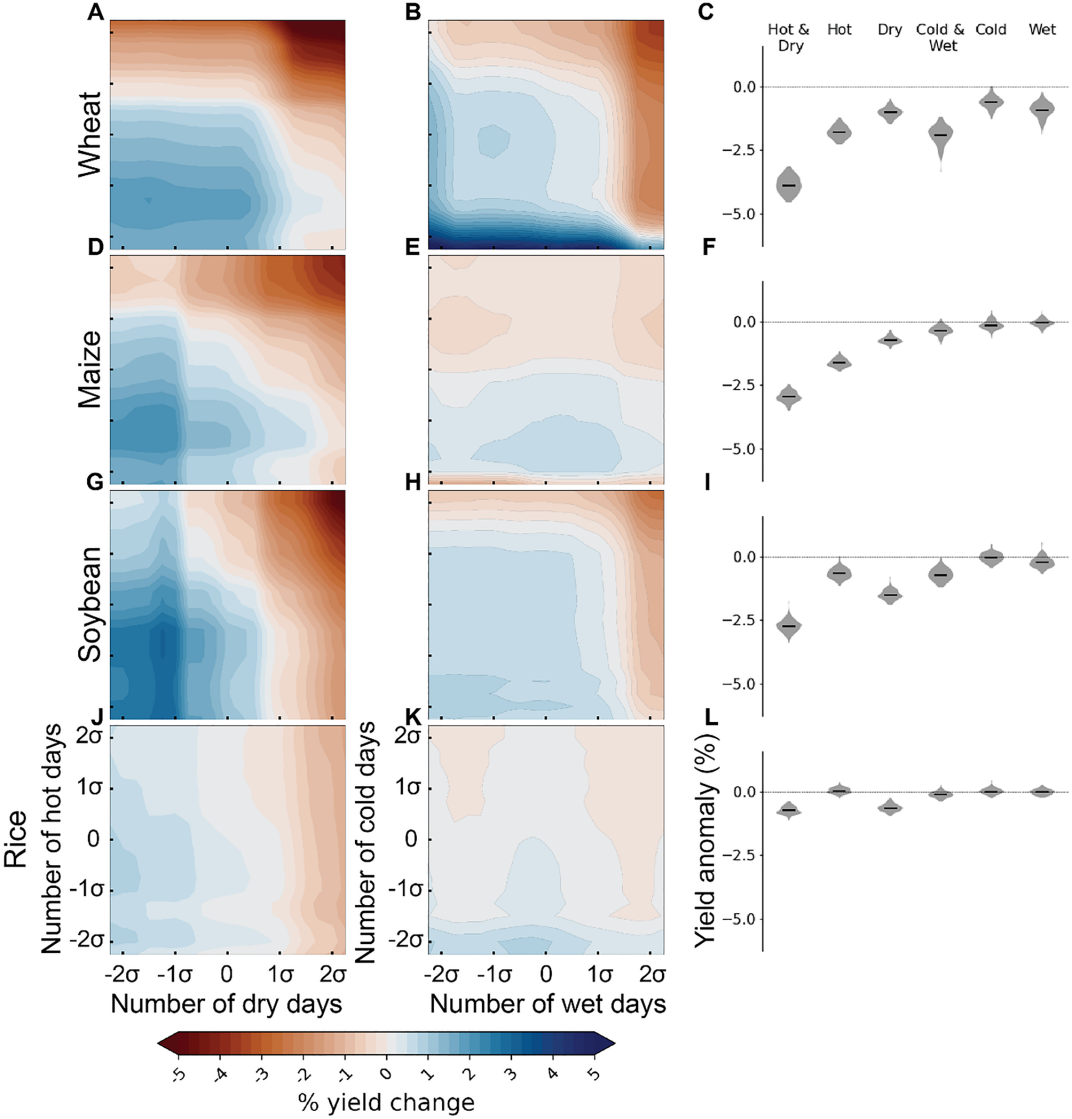
Global results about the relationship between crop yield anomalies (%) and the number of hot, and dry (cold, and wet) days during the growing season. Results are shown for studied crops: wheat (**A**,**B**,**C**), maize (**D**,**E**,**F**), soybean (**G**,**H**,**I**), and rice (**J**,**K**,**L**). Here, the number of hot, and dry (cold, and wet) days refer to days during the growing season above 90th (below 10th) percentile for temperature and soil moisture deficit, respectively (i.e. the extreme indicators are calculated separately for each growing season). The results are based on partial dependencies calculated separately for each extreme scenario from the XGBoost models trained at global scale (see “Methods”). Uncertainty of the results was evaluated by calculating the partial dependencies 100 times while sampling the years in each training and validation set. The violin plots describe the range of estimated crop yield anomalies across model runs in a situation where the respective weather anomalies are fixed at 1.5σ, while the heatmaps describe averaged impacts across a range of hot, and cold (cold, and wet) scenarios.

The trained XGBoost models then allowed us to study the relationship between extreme weather conditions and crop yield anomalies globally. Here, the prevalence of extreme conditions was calculated as the number of days during the growing season in the hottest, driest, coldest, and wettest 10% category, respectively (see “Methods”). Based on partial dependencies calculated from the globally trained models (see “Methods”), we observed largest yield reductions when both heat and drought prevail during the growing season for all studied crop types (Fig. 2). Although globally not as influential for the model output (Fig. S6), cold and wet conditions were also often observed to reduce crop yields at global scale (Fig. 2).

**Figure 3:**
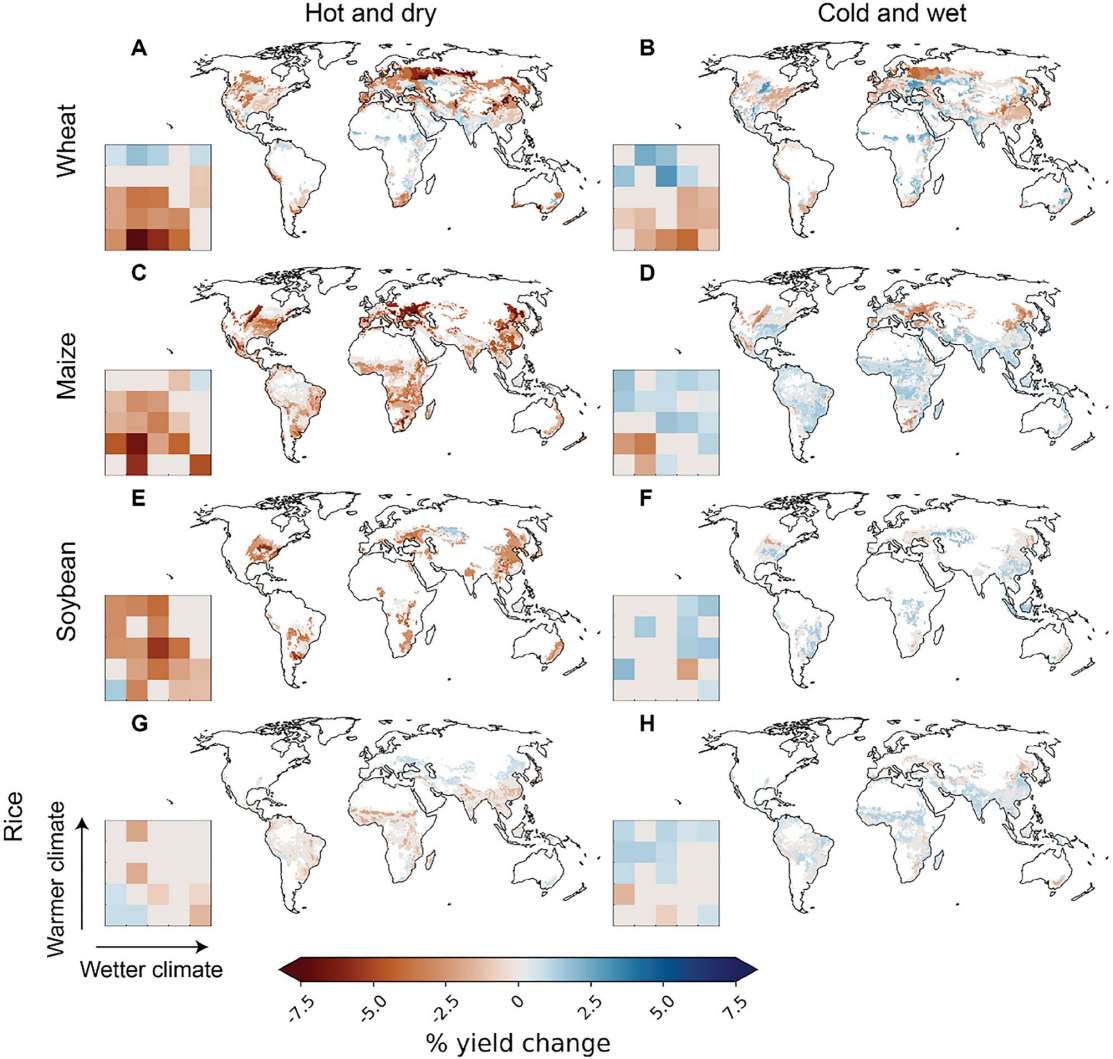
The relationship between crop yield anomalies (%) and co-occurring hot-dry and cold-wet conditions during the growing season for each climate bin. Results are shown for studied crops: wheat (**A**,**B**), maize (**C**,**D**), soybean (**E**,**F**), and rice (**G**,**H**). See map and division of the climate bins for each crop in Fig. S2. Here, the number of hot, and dry (cold, and wet) days refer to days during the growing season above 90th (below 10th) percentile for temperature and soil moisture deficit, respectively (i.e. the extreme indicators are calculated separately for each growing season). The results, which are based on partial dependencies calculated from the XGBoost models trained for each climate bin separately (see “Methods”), estimate crop yield anomalies in a situation where the number of hot and dry days deviates 1.5σ from the long-term average. Uncertainty and confidence intervals of the results were evaluated by calculating the partial dependencies 100 times while sampling the years in each training and validation set. The mapped value is the average of the 100 crop yield anomaly estimates obtained. However, if zero was within the 95% confidence interval of the crop yield anomaly estimates, the mapped yield anomaly estimate was also set to zero. The maps presented in the figure were created with Matplotlib and Cartopy Python modules^38–40^.

Out of the crops studied, wheat showed the strongest sensitivity to co-occurring climate extremes at the global level (Fig. 2A,B,C). For co-occurring hot-dry conditions (i.e., when the number of hot days, and dry days during the growing season both deviate 1.5σ from the long-term average), we estimated globally an average − 3.9% (95% confidence interval: − 3.3% to − 4.5%) reduction in crop yield. Here, the confidence intervals of the global results were estimated by evaluating the impacts 100 times while sampling the years in each training and validation set (see “Methods” for details). In many climate bins, however, much larger reductions were observed (Fig. 3A,B). For cold-wet events, the global average reductions were slightly lower (− 1.3% to − 2.7%), and comparable to individually occurring heat (− 1.3% to − 2.2%) and drought (− 0.7 to − 1.4%). Also, individually occurring wet conditions reduced yields (− 0.1% to − 1.0%), which aligns with a previous study that reports lower wheat yields during wet conditions in humid regions^41^. Based on the models trained for each climate bin, wheat yield reductions for co-occurring heat and drought were larger in relatively cool regions, located in, for example, Russia and China (Fig. 3A,B), which also showed relatively strong relationship between crop yield anomalies and weather generally (Fig. 1B).

Maize yields were found to be globally − 3.0% (− 2.6% to − 3.4%) lower when hot and dry conditions co-occur (Fig. 2D,E,F). The yield reductions were larger for hot (− 1.3% to − 1.9%) conditions, compared to dry (− 0.4% to − 1.0%) or to cold-wet (0.0 to − 0.7%) conditions. The difference between the impacts of hot and dry events might be due to maize being a C4 plant, and hence more tolerant to drought compared to C3 plants^42^. Hot-dry conditions reduced maize yields in almost all climate bin specific models, with largest reductions observed geographically for example in East Asia and eastern Europe (Fig. 3C,D), which aligns with a previous understanding^25^. Interestingly, cold-wet conditions increased maize yield during cold-wet conditions in many, especially relatively warm, climate bins (Fig. 3C,D).

Also, for soybean yield, co-occurring heat and drought had the largest impacts (a reduction of − 2.3% to − 3.2%; Fig. 2G,H,I), while cold-wet events had a lesser negative effect (− 0.3% to − 1.1%) comparable to individually occurring heat (− 0.3% to − 1.0%) and drought (− 1.2% to − 1.8%). Spatially, hot-dry events reduced soybean yields for example in North and South America as well as eastern Asia (Fig. 3E,F)—all highly important regions for global soybean production. Noteworthy, for North America, also previous research has reported yield reductions during compound hot-dry events^43^.

Although the general patterns were similar compared to the other crop types studied, rice showed the smallest sensitivity to the climate extremes studied here. On average, we estimated co-occurring heat and drought to reduce rice yield by − 0.7% (− 0.4% to − 1.0%), while cold-wet conditions were not observed to have any impact (Fig. 2J–L). Compared to the other crop types, rice showed more uncertainty when the responses were estimated across models trained for each climate bin also for hot-dry conditions (Fig. 3G,H). The mixed-signal pattern observed for different climate bins is probably due to the generally weak relationship between climate variations and rice yield anomaly variability (Fig. 1).

The results for hot-dry impacts remained consistent when the analyses were conducted with different growing season set-up, soil moisture data and machine learning algorithm as well as with including only extreme indicators as model predictors, and irrespective whether the data for extreme conditions were temporally de-trended or not (Figs. S7–S12). However, when a different crop yield dataset^44^ was utilized for training the model, some differences exist (Fig. S13). These differences are potentially due to different resolution of the crop yield statistics utilized to derive the respective data sets. The reference data are based on crop yield statistics collected at the country level^44^, while the crop yield data utilized here are collected from approximately 20,000 political units^27^. Although, it should be noted that also with these data, co-occurring hot-dry conditions were generally shown to reduce yields.

### Increasing frequency of heat and drought events during the growing season

**Figure 4:**
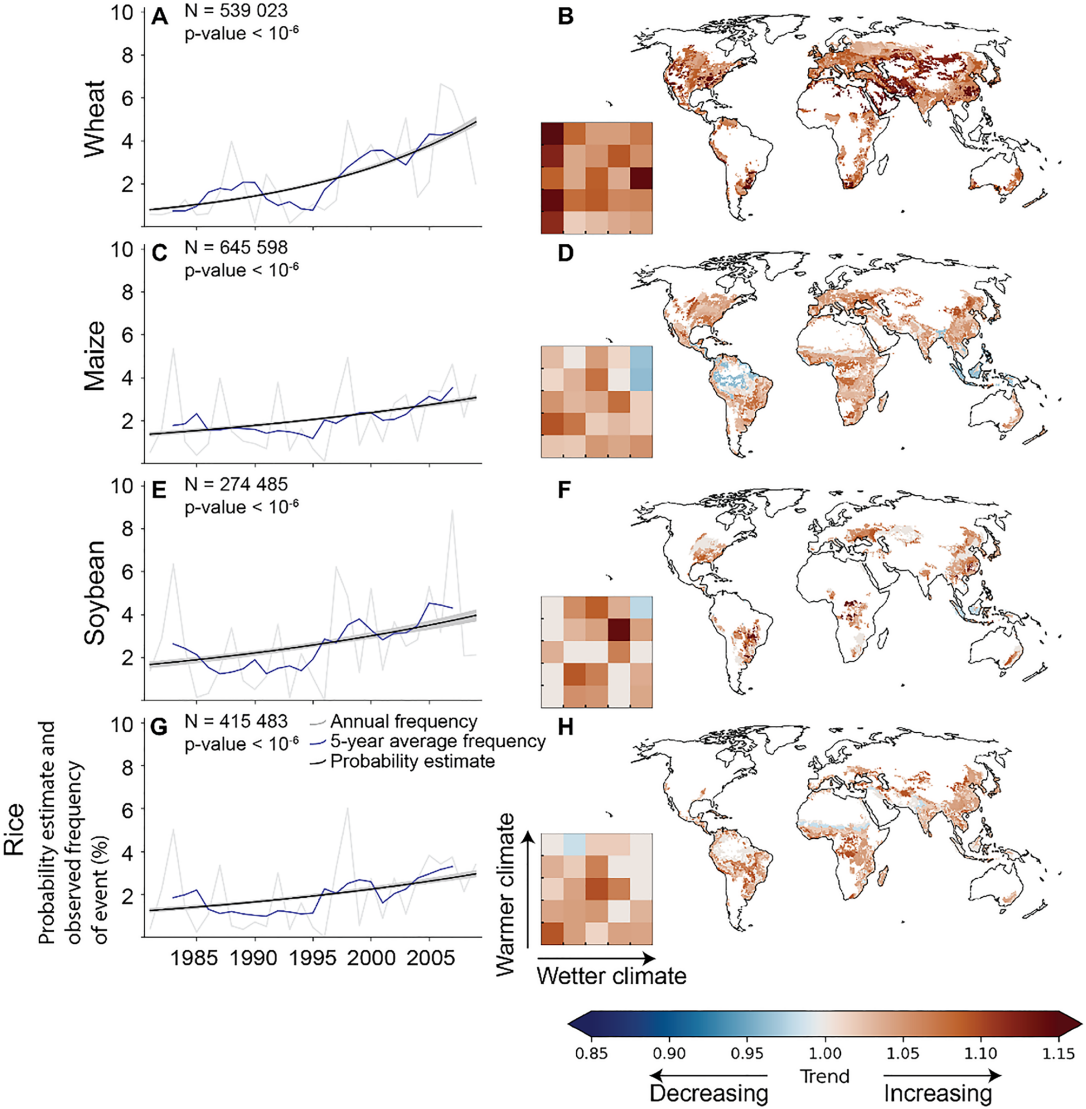
Historical evolution in the probability of co-occurring hot and dry conditions during the growing season between 1980 and 2009. Results are shown for studied crops: wheat (**A**,**B**), maize (**C**,**D**), soybean (**E**,**F**), and rice (**G**,**H**). Here, a grid cell is considered hot and dry for a specific year, if the number of both hot, and dry days during the growing season deviates at least 1.5σ from the long-term average. The number of hot, and dry days refer to days during the growing season above 90th percentiles for temperature and soil moisture deficit, respectively (i.e. the extreme indicators are calculated separately for each growing season). The historical evolution of the probability in co-occurring hot-dry events was assessed by logistic regression. For the global results (**A**,**C**,**E**,**G**), event frequencies were calculated as a percentage of hot-dry events for each year and as a five-year average, whereas the uncertainty intervals for the regression lines in the figure were calculated by bootstrapping (N = 100) the observations and plotting the regression line for each sample (in gray color). For the climate bins (**B**,**D**,**F**,**H**), the trend obtained from the logistic regression are represented as odds ratios. If odds ratio is above (below) one, the trend is increasing (decreasing). Note that, if the trend was statistically insignificant (*p-value* > 0.05) for any of the statistical tests conducted (see “Methods”), the reported odds ratio was set to one (i.e., no trend). The maps presented in the figure were created with Matplotlib and Cartopy Python modules^38–40^.

We showed above that co-occurring drought and heat during the growing season is linked to reduced crop yields across the studied crops (cf. Figs. 2, 3). To assess whether the occurrence of these extreme events, and thus their potential impact on crop yields, has changed over the past decades, we calculated how their probability has evolved during the study period. Namely, we used logistic regression to analyse how the co-occurrence of hot-dry (and cold-wet) conditions have changed from 1980 to 2009. To do that, we first coded a year in a grid cell as extremely hot and dry, if the number of both hot days, and dry days was above 1.5σ compared to the long-term average. The regression was then calculated globally as well as for each climate bin separately considering each year-grid cell pair as an observation. Our analysis shows that at the global level there was a significant increasing trend in co-occurring hot and dry conditions for all crops during 1980–2009 (Fig. 4).

Wheat showed globally the strongest increase in co-occurring hot and dry conditions (Fig. 4A,B). We estimated that, for any grid cell within the global wheat production regions, the probability of co-occurring hot and dry conditions increased from less than 1% in the early 1980s to ~ 5% in 2009. The observed trends were consistent also when using another soil moisture data (GLEAM^45^), although smaller (Fig. S14), probably because of a non-existent (*p*-value > 0.05) trend in solely dry conditions (Fig. S17). For those climate bins, where a trend was observed (Fig. 4B), the patterns remained similar compared to the global trends (Fig. 4A,B, Fig. S14A,B), southern Africa, eastern China and the Middle East showing the strongest increases in co-occurring hot-dry conditions.

For maize, soybean, and rice, the trends were relatively similar, and there has been approximately a twofold increase in the probability of co-occurring heat and drought during the study period (Fig. 4). For soybean, and rice, the global trends were weaker, although still statistically significant (*p*-value < 10^–5^ for both), when the analyses were conducted utilizing the GLEAM soil moisture data (Fig. S14). For maize, we did not observe a consistent trend in co-occurring heat and drought (*p*-value > 0.05) when utilizing the GLEAM soil moisture data. Generally, the trends for different climate bins aligned with those observed at the global level (Fig. 4). However, for a few climate bins, the probability for co-occurring heat and drought seemed to decrease during the study period. For cold and wet conditions, the trends were consistently decreasing for all the studied crop types irrespective of the data used (Figs. S15, S16, S18).

It should be noted that, although, climate change is likely a major driver behind these trends, they are also subject to a range of other anthropogenic and natural influences^46,47^. For example, irrigation decreases soil moisture deficit and can also lower temperature irrespective of the weather conditions^48,49^, aerosols are also known to decrease temperatures, especially in some highly populated regions of the globe^50^.

## Discussion

Using reported crop yield statistics collected across 20,000 political units, we show that co-occurring hot and dry events have globally consistent negative effects on wheat, maize, soybean, and rice yields (cf. Fig. 2). Extremely cold and wet conditions were observed to reduce crop yields globally too, although to a lesser extent and the impacts being more uncertain. These results extend existing global studies, which have mainly studied individually occurring extremes or utilized national level data^2,13,15,21,26^. Furthermore, our analyses show, that the probability of co-occurring heat and drought during the growing season increased globally between 1980 and 2009 for all studied crops. This aligns with previous estimates, which have shown that the proportion of crop growing areas affected by extreme weather events has increased^46,51^. Noticeably, consistent increasing trends in co-occurring heat and drought were observed in climate bins located in northern latitudes (cf. Fig. 4), for example in Europe and North America, where agriculture is sometimes viewed to potentially benefit from climate change (for example, through longer growing seasons)^52^. However, our analyses show that these potential benefits may be offset by the increasing probability of hot and dry weather extremes.

Although cold and wet events were also observed to reduce crop yields globally, their impacts were more uncertain and diverse compared to those of heat and drought. At the climate bin level, cold-wet events were often observed not to have any influence or even increase crop yields, especially in warm climates. This could partially be because cold anomalies, defined relative to local climate, are also related to reduced heat stress and evaporative demand which can benefit crop growth. This is the case also for abnormally wet growing seasons, which could be beneficial for crops in normally water-limited conditions. These issues in the analyses could potentially be overcome by using absolute thresholds for defining the extreme weather indicators. However, the weather conditions that agriculture has adapted to vary geographically, which would bring challenges to defining global thresholds. For example, a 0 ºC threshold, based on water’s freezing point, might be sensible in cool regions, but less useful in the tropics.

The extent of global crop yield anomaly variability explained by weather was relatively low when using a crop yield data set, based on national level crop yield statistics disaggregated with satellite-based net primary productivity^44^. Although co-occurring heat and drought was also observed to generally reduce crop yields with this data set, the results were noisier especially for the impacts of cold and wet events (Fig. S13). It should be noted that some differences between results are expected, as the two crop yield data sets utilized in this study have been shown to not correlate in a large proportion of global croplands^4^. It might also be that the sub-nationally reported data used in the main analyses captured impacts of spatially more granular weather events; this is especially beneficial for large countries, such as the United States and China, where weather conditions might largely vary in distinct parts.

To keep the data consistent throughout the different parts of the study and considering the uncertainties related to adaptation, we did not de-trend the extreme climate indicators for the main analyses investigating their impacts on crop yields. Another source of uncertainty is that we studied weather impacts on crop yields in a fixed 90-day period before harvest. Although these 90-day periods were defined for each grid cell separately (see “Methods”) and these periods cover the crop development stages most sensitive to stress^10,53^, extreme conditions occurring during earlier phases of crop development can also have major influence on yields^54^. However, the observed impacts remained the same also with de-trended extreme indicators and when calculating the climate indicator for the whole growing season (cf. Figs. 2, S8–S9).

Our findings show that the probability of hot and dry weather extremes has increased in the study period (Fig. 4). This finding does not necessarily imply that effects on crop yield have also increased. More frequent hot and dry events point to increased hazard, but crop yield is also determined by changes in the exposure of crops and changes in the vulnerability to heat and drought events. It is well known, for example, that the growing areas of the crops considered in this study have changed considerably in the study period with increasing crop shares of maize and soybean in temperate climate regions^55^. Together with changes in sowing and harvest dates this results in a modified exposure of these crops to heat and drought^56^. In addition, there are efforts to adapt crop production to more frequent extreme weather, for example by breeding heat and drought tolerant cultivars and by improving crop and soil management. However, an analysis of the impact of these complex interactions on crop yields was beyond the scope of the present study.

Future research could investigate how the daily co-occurrence of extreme weather impact crop yields, as recent research conducted in growth chambers suggests that the impacts of co-occurring heat and drought become even larger, when inspected at daily level^57^. This approach would be very interesting, as heat and drought can amplify each other in the environment. Higher temperatures lead to larger evaporation, which dries the soil. Drier soils can then lead to higher temperature close to the plants, if the water available for evapotranspiration and the related cooling effects decrease.

A clear understanding of climate-induced food shocks is imperative in striving for food security for all. Our results provide an important step towards understanding the effects of co-occurring extreme events on global food crop production. As the probability of crop yield reducing dry and hot weather has increased, global efforts should help farmers to adapt to weather extremes in addition to reducing the emissions that cause climate change.

## Materials and methods

### Crop yield data

For crop yield data (t ha^−1^), we used reported annual maize, rice, soybean, and wheat yield data collected across approximately 20,000 political units^27^. The current version of the data span years 1961–2013 and we use it with a resolution of half degree (~ 50 km at the equator). Here we use data only for years 1981–2009, due to the restrictions posed by the climatological data sets (see below). Gaps that exist in the collected crop statistics in specific countries were filled with the last time period available 5-year subnational average and finally scaled to match UN FAO reported numbers at the country level. It should be noted that the gaps are concentrated in the earlier time-period of the data, which is not used here. The crop yield data used in this study have been previously used in numerous studies on historical crop yield and hydrometeorological variability^1,2,27,47^.

Furthermore, for reference, we also used another global gridded crop yield data set developed by Iizumi and Sakai (2020)^44^. These data are based on country-level crop statistics, which are disaggregated to 0.5º resolution using satellite-based net primary productivity estimates and spans 1981–2016 (data for 1984–2009 is utilized here). These data have also been used extensively in global studies about drivers of interannual crop yield variability^58,59^.

To remove temporal trends related to for example changes in management conditions, and thus to isolate interannual variability of crop yield anomalies, for each raster cell the crop yield data were de-trended. The de-trending was conducted by subtracting a five-year moving average from the annual values, similarly to several previously conducted studies about yield variability^4,58^. The anomalies were then divided by five-year averages to obtain proportional annual deviation from the normal values. Previous studies have tested other de-trending methods as well but have not found the selected method to have a major influence^4,59^.

### Temperature and precipitation data

For temperature (ºC) and precipitation (mm day^−1^) data we used the AgMERRA re-analysis data set that fuses modelled weather reanalysis with observations to more accurately capture extreme weather events for agricultural studies^28^. AgMERRA provides daily estimates of minimum and maximum temperature, precipitation, solar radiation, and humidity at 0.25 degree resolution for years 1980–2010. It is bias-corrected especially for agricultural areas. Here, we used data for daily average, minimum and maximum temperature as well as daily precipitation. The AgMERRA data set has been used extensively for modelling climate impacts on agriculture, including in The Agricultural Model Intercomparison and Improvement Project (AgMIP)^60^.

To align with the other data sets used here, the temperature and precipitation data were resampled from 0.25 to 0.5 degree resolution using linear interpolation. Further, for obtaining an estimate of the daily temperature distribution, the daily data were interpolated assuming that the daily temperature distribution follows the sine function, and using minimum temperature as the lowest value and half of the difference between minimum and maximum temperature as amplitude.

### Soil moisture deficit data

The soil moisture data (m^3^/m^3^) for this study come from ERA5 re-analysis product spanning from 1979 onwards^29^. The ERA5 dataset provides hourly estimates of soil moisture (as well as a wide range of other hydroclimatological parameters) at ~ 0.28 degree (interpolated linearly here to 0.5 degree resolution) resolution at global scale. It is based on the Integrated Forecasting System (IFS) Cy41r2 and maintained by European Centre for Medium-Range Weather Forecasts (ECMWF). Here, we used hourly soil moisture obtained at 12:00 pm UTC as a daily soil moisture estimate. In ERA5 soil moisture is estimated by utilizing Advanced Scatterometer (ASCAT) soil moisture data combined with other land surface variables (including e.g. temperature and humidity) via a simplified point‐wise Extended Kalman Filter^61^.

We acknowledge that there are uncertainties in global soil moisture estimates. Therefore, for reference, we also obtained soil moisture data from another source, namely from the Global Land Evaporation Amsterdam Model (GLEAM) v3.2a^45^. In GLEAM, surface soil moisture is estimated based on microwave satellite observations combined with a water balance model. GLEAM provides data for both surface and root-zone soil moisture, and here surface soil moisture data are used.

Because soil conditions vary across regions, the soil moisture data were standardized and transformed to relative soil moisture deficit. This transformation was conducted by subtracting each daily value from the maximum reported daily soil moisture value of the whole data set and dividing with the difference between maximum and minimum values. Hence, in these transformed soil moisture data, larger values mean drier conditions (i.e. larger soil moisture deficit).

### Growing season data

The growing season data (i.e. planting and harvest dates) were obtained from AgMIP^4,60^. In AgMIP, the planting and harvest dates are determined based on three sources: MIRCA2000^35^, Sacks et al. (2010)^62^, and those simulated by the Lund-Potsdam-Jena managed Land (LPJmL) model^63^. Planting and harvest dates are separately estimated for irrigated and rainfed scenarios. In these data, for those areas where data from MIRCA2000 or Sacks et al. exist, the values closest to the LPJmL estimates are selected, while the modelled LPJmL estimates are used elsewhere. Because crops tend to be most susceptible to weather extremes around the time of harvest, the time period from 90 days prior and until harvest were selected for the main analyses here. The 90-day time interval before harvest was also chosen because the seasonal weather variation during the growing season can be large, as is the case for example for winter wheat. Note still, that we conducted the same analyses also using climate indicators calculated for the full growing season (Table S1).

### Crop-specific climate bins

We developed 25 crop-specific climate bins based on growing season average temperature and annual precipitation^28^. The binning divides crop-specific growing areas into 25 climatologically analogous zones, which have similar annual precipitation and temperature characteristics. For each crop, the binning was conducted in two steps. First, we divided considered grid cells with data for the crop in questions into quintiles based on growing season temperature. After that, each temperature quintile was again divided into quintiles based on total annual precipitation.

### Climate indicators

To obtain annual growing season-specific temperature and soil moisture deficit distributions for each crop type and raster cell, the daily values were allocated to bins, which for soil moisture range from 0.0 to 1.0 (0.001 intervals) and for temperature from -20 to 60 ºC (0.1 ºC intervals). Hence, the unit for these aggregated data is days per soil moisture or temperature bin. Practically, we obtain a histogram for growing season temperature and soil moisture conditions for each grid cell and year. Because the growing season data are provided for irrigated and rainfed areas separately and a single grid cell can include both irrigated and rainfed fields, we calculated a harvested area weighted average number of days per bin of the irrigated and rainfed growing season scenarios. Here, the harvested areas data were obtained from MIRCA2000^35^. These binned daily data were then transformed into explanatory variables by calculating percentiles of the respective histograms. Specifically, for each raster cell we calculated the days below 10th (cold, and wet indicators), and above 90th (hot, and dry indicators) percentiles (Figs. S19–S20) for temperature and soil moisture deficit across all years. Note, that here each extreme indicator was calculated separately.

In addition to the variables describing extremely hot, dry, cold, and wet conditions, we used additional weather and climate variables to describe average conditions during the growing season (Table 1). These variables were included to account for potential interactions between the average and extreme conditions. For the main analyses, we used average growing season temperature, and soil moisture as well as total yearly (prior to harvest) and growing season precipitation as explanatory variables in the model. The full list of model-set ups conducted for this study can be found in Table S1.

After the aforementioned steps, all the data were standardized by calculating their z-score (by subtracting the mean and dividing with standard deviation) prior to running the statistical analyses. To ensure an even distribution of observation across the range of inspection, the number of dry and wet days during the growing season were transformed by calculating their square root. Finally, all those climatological data describing average conditions were linearly de-trended (see correlation matrices across the climate variables as well as crop yield anomaly in Fig. S21 and the relationship between the extreme indicators in Fig. S22). Please note that, for comparison, we performed the main analyses of this study also for a scenario where also the extreme indicators were de-trended.

### The effects of heat and drought on crop yields

To model interannual crop yield variability based on the above-described explanatory variables, we used XGBoost regression. XGBoost regression is a non-parametric machine learning method based on boosted decision trees. In XGBoost an ensemble (here 400 trees with a maximum depth of 3) of decision trees are trained iteratively^31^. The procedure of using previously calculated errors in each iteration step, improves performance and can effectively avoid the overfitting problem associated with individual decision trees. Further, because XGBoost utilizes decision trees for training and prediction, it does not make assumptions about the distribution of the data or the independence of the explanatory variables and is relatively robust to outliers. Because XGBoost regression is a non-parametric regression method and able to capture non-linear dependencies, we expect it to perform relatively well for modelling the relationship between crop yields and climate variability^64^. For comparison, the analysis was also conducted with Random Forest regression (Fig. S10).

Here, the XGBoost model was fitted for all crop types (N = 4) separately at the global level as well as for each climate bin (N = 25), considering each year-cell combination as an observation. Prior to fitting the model, all years were randomly split into four groups. For each of these groups, crop yield anomalies were estimated with the XGBoost model using data from the other three groups for model training (i.e. we obtain out-of-sample predictions for each grid cell and year). When training each model two of the three training groups were utilized for fitting the model whereas one group was utilized for evaluating training progress. Namely, to avoid overfitting, the training of each XGBoost model was halted when the evaluation error increased for 40 consecutive training rounds.

Furthermore, we assessed the uncertainty associated with XGBoost hyperparameters by conducting a randomized grid search each time a model is trained. In the randomized grid search (5 iterations), we sample four regularizing hyperparameters: the proportion of training data (50–100%), and the number of variables used (50–100%) for building each tree, as well as the lambda (0.5–1.5) and gamma (0–0.05) parameters. Based on the 5 iterations, we select the most suitable set of hyperparameters using a threefold cross validation, where we ensure that each fold has data for different years. Although this method will often not provide the most optimal set of hyperparameters, it does allow us to assess the sensitivity of our results to the hyperparameters.

After the training and prediction procedure was conducted for all four groups, we obtained a continuous time series of estimated crop yield anomaly for each grid cell. We used the squared Pearson’s correlation coefficient between reported and estimated crop yield anomalies aggregated (with a harvested area-weighted average) to global and climate bin level to evaluate the explanatory power of the model. To assess uncertainty related to the grouping of the years, this operation was conducted 100 times by randomly splitting the years into the four groups in each iteration. To assess the importance of each climate variable in the prediction of crop yield anomalies, we calculated and combined the Shapley values across all the random splits (Fig. S6)^65^.

To understand the effects of hot and dry as well as cold and wet conditions on crop yields, we calculated the partial dependence of the trained XGBoost models. This was conducted by restricting the values in hot, dry, cold, and wet conditions, while randomly selecting a sample of 1000 observations for all other variables and then calculating the output of the model. The calculation of the partial dependence was conducted using the ‘_partial_dependence_brute’ function in the ‘sklearn’ Python library^66^. Since, this method calculates the marginal effects of one or two variable(s) by taking a random sample of the other variables, it is possible that the results become unstable, if the combinations are very unlikely. To account for this potential instability, and to assess the uncertainty related to the grouping of the years, the partial dependence calculations were conducted for each of the 100 random splits. For each iteration round, the partial dependencies are averaged over the four train-test splits, and hence we obtain 100 estimates of crop yield anomaly for each extreme climate scenario.

More specifically, partial dependence was calculated as the output of the trained model by varying the anomaly in hot, dry, wet, and cold conditions (from − 2.25 to 2.25 with 0.25 intervals). For example, the partial dependence of crop yield to solely dry conditions (i.e. when the number of dry days deviates 1.5σ from the long-term average) was calculated by selecting a random sample of 1000 observations from the training data, and switching the value 1.5σ to the drought column of all observations and calculating the average model output with this data. Similarly, the partial dependence of co-occurring dry and hot conditions (i.e., when the number of hot days, and dry days during the growing season both deviate 1.5σ from the long-term average) was calculated by switching the value 1.5σ to both heat and drought columns of all observations.

### Historical changes in co-occurring extreme weather conditions during the growing season

The trend in the probability of co-occurring hot and dry (wet and cold) extreme events was assessed with logistic regression utilizing the ‘statsmodels’ Python library^67^. The regression was calculated globally and separately for each climate bin considering each year-grid cell pair as an observation. The data were transformed into logical event space prior to running the regression. Namely, a year in a grid cell was considered hot and dry (cold and wet) if the standardized anomaly in number of both hot and dry (or cold and wet) days during the growing season was at least 1.5σ. The same trend assessment was also conducted for solely dry, hot, cold, and wet events at the global level.

As logistic regression estimates the probability of an event with the logit function, for the global analyses we transformed the logit estimates to probability, while for the climate bins, the logit estimates were transformed to odds ratio to provide a single value describing the trend. The statistical significance of the logistic regression models was assessed with the Likelihood ratio test, while the statistical significance of the coefficients was tested with two distinct methods. Firstly, the coefficient *p-values* were calculated parametrically with the two-tailed t-statistic, and secondly non-parametrically by bootstrapping (N = 100) the observations and calculating the regression coefficient for each sample. The results were considered statistically significant if the *p-values* of all these three tests were below 0.05. Finally, the linearity of the globally aggregated log-odds was visually checked by calculating average log-odds values of co-occurring dry and hot (or cold and wet) events for each temporally defined quintile (Figs. S23–S24).

## Supplementary Information

Supplementary Information.

## Data Availability

This work was built upon publicly available datasets. Nevertheless, the crop yield data are available from D.K.R. upon a reasonable request, whereas the other data utilized to conduct this study are available from M.H. upon a reasonable request.

## Code availability

The processing codes are available at https://github.com/matheino/crops_vs_extremes.
