# Supplementary material for: Increased probability of hot and dry weather extremes during the growing season threatens global crop yields
Source: Sci Rep. 2023 Mar 3;13:3583. doi: 10.1038/s41598-023-29378-2 (PMC9984494; doi:10.1038/s41598-023-29378-2)
Supplement: Supplementary file 1 — Supplementary Information. [file 41598_2023_29378_MOESM1_ESM.pdf]

Supplementary Information for

Increased probability of hot and dry weather extremes during the growing season threatens global crop yields

Matias Heino, Pekka Kinnunen, Weston Anderson, Deepak K. Ray, Michael J. Puma, Olli Varis, Stefan Siebert, Matti Kummu

Corresponding authors: Matias Heino, Matti Kummu
Emails: matias.heino@aalto.fi, matti.kummu@aalto.fi

Table S1. The modeling set-ups conducted in this study.

Variable	Main	Fig. S7	Fig. S8	Fig. S9	Fig. S10	Fig. S11	Fig. S12	Fig. S13
**Hot days**	AgMerra[1]	AgMerra	AgMerra*	AgMerra**	AgMerra	AgMerra	AgMerra	AgMerra
**Dry days**	ERA5[2]	GLEAM[3]	ERA5*	ERA5**	ERA5	ERA5	ERA5	ERA5
**Wet days**	ERA5	GLEAM	ERA5*	ERA5**	ERA5	ERA5	ERA5	ERA5
**Cold days**	AgMerra	AgMerra	AgMerra*	AgMerra**	AgMerra	AgMerra	AgMerra	AgMerra
**Average temperature**	AgMerra	AgMerra	AgMerra	AgMerra**	AgMerra	AgMerra	-	AgMerra
**Average soil moisture**	ERA5	GLEAM	ERA5	ERA5**	ERA5	ERA5	-	ERA5
**Average precipitation (growing season)**	AgMerra	AgMerra	AgMerra	AgMerra**	AgMerra	AgMerra	-	AgMerra
**Average precipitation (annual)**	AgMerra	AgMerra	AgMerra	AgMerra**	AgMerra	AgMerra	-	AgMerra
**Climatological growing season temperature**	-	-	-	-	-	AgMerra	-	-
**Climatological precipitation**	-	-	-	-	-	AgMerra	-	-
**Crop yield anomaly**	Ray et al. (2019)[4]	Ray et al.	Ray et al.	Ray et al.	Ray et al.	Ray et al.	Ray et al.	Iizumi and Sakai (2020)[5]
**Model**	XGBoost[6]	XGBoost	XGBoost	XGBoost	Random Forest[7]	XGBoost	XGBoost	XGBoost

*) Extreme indicator de-trended
**) Indicator calculated for full growing season

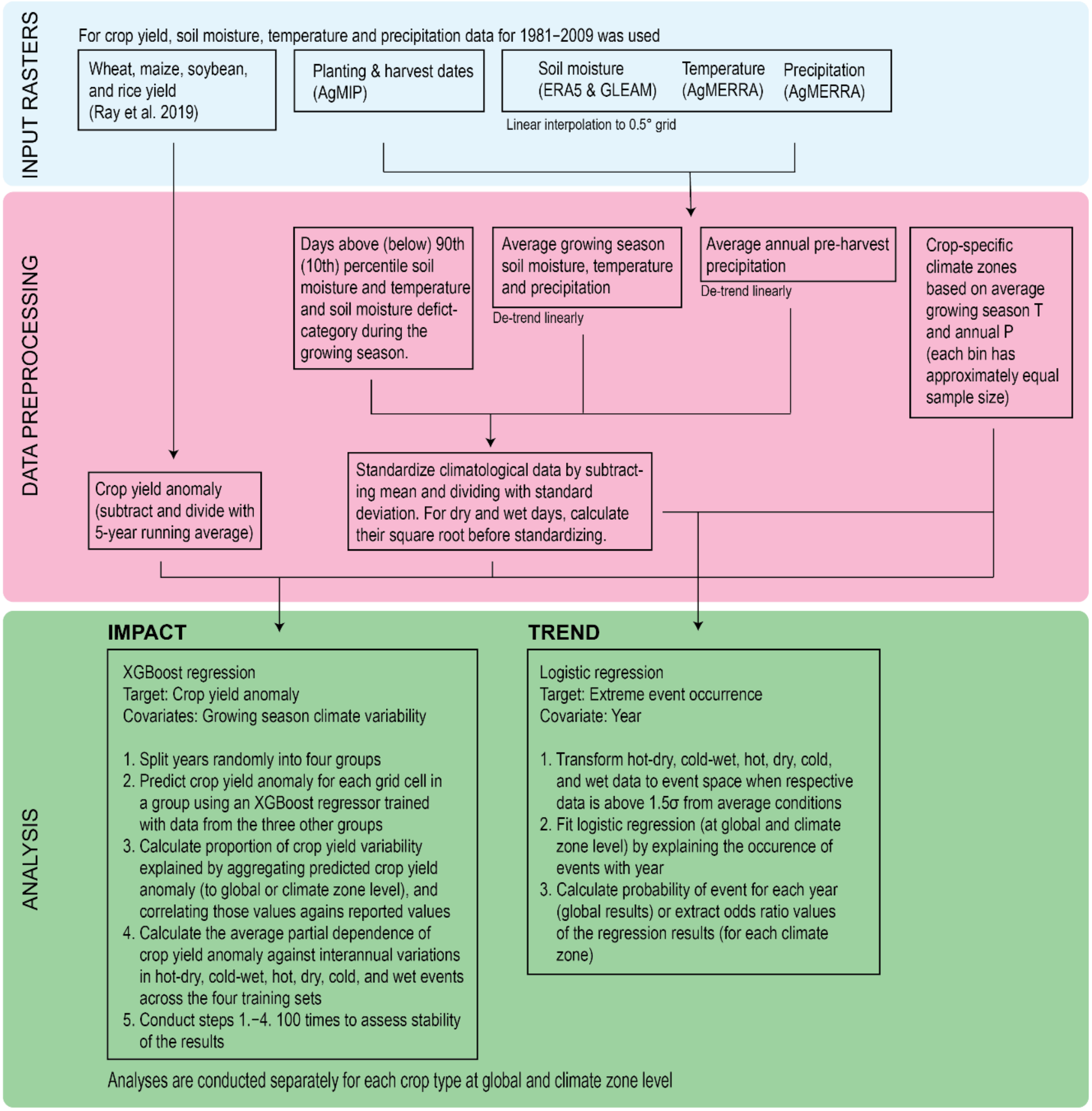

Fig. S1. Methodological framework of the study.

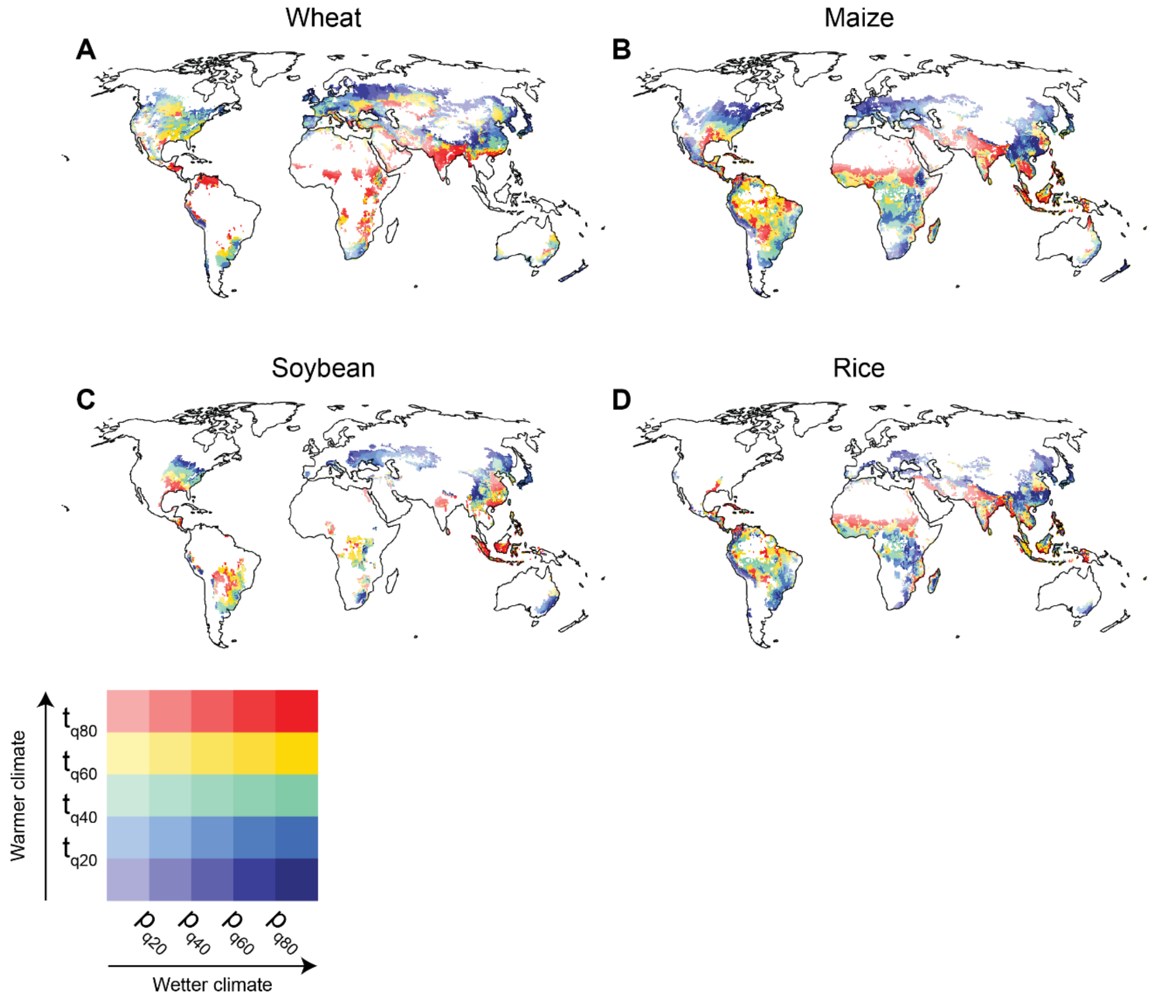

Fig. S2. Crop-specific climate zones for wheat (A), maize (B), soybean (C), and rice (D). The zoning is based on growing season mean temperature and total annual precipitation (1). For each crop, the zoning was conducted in two steps. First, all considered grid cells were divided into quintiles based on growing season temperature. After that, each temperature quintile was again divided into quintiles based on annual precipitation (see Methods for more details).

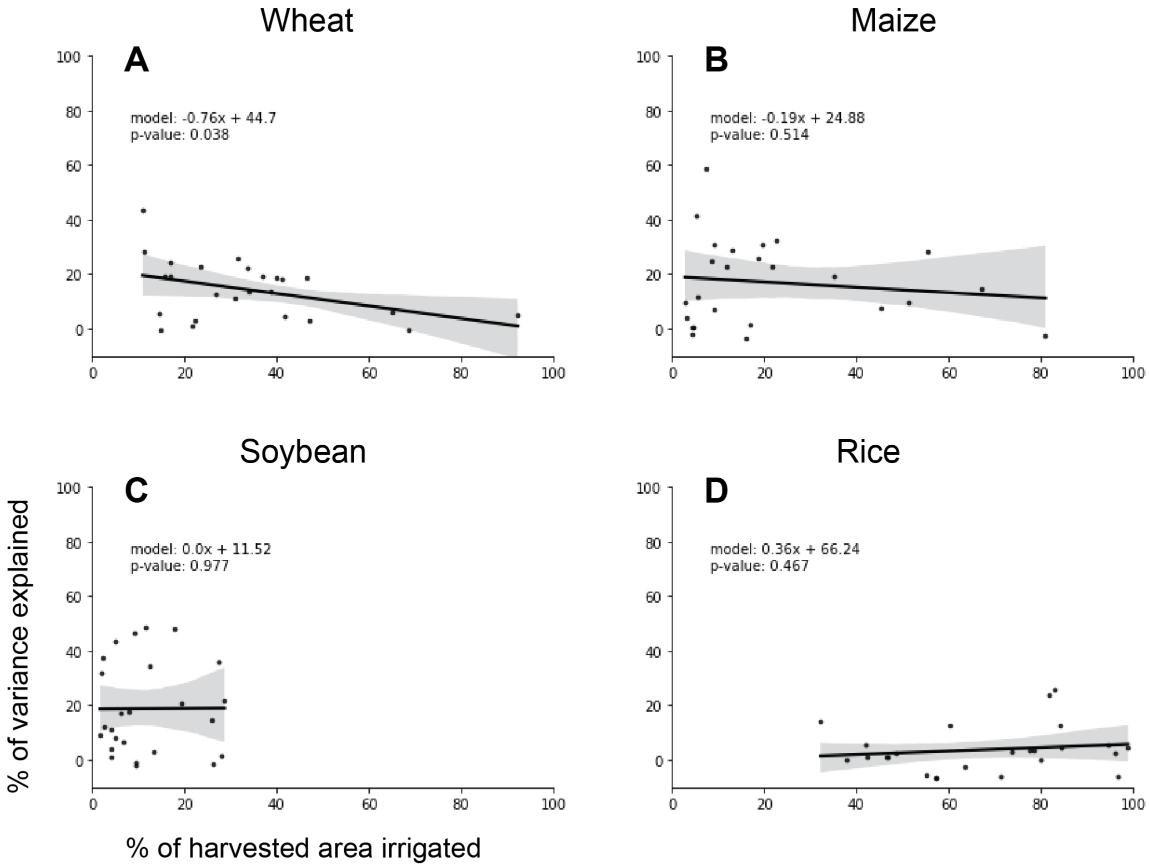

Fig. S3. The relationship between proportion of irrigated areas[8] (2) and proportion of wheat (A), maize (B), soybean (C), and rice (D) yield variance explained by climate variability. Each point represents one climate zone.

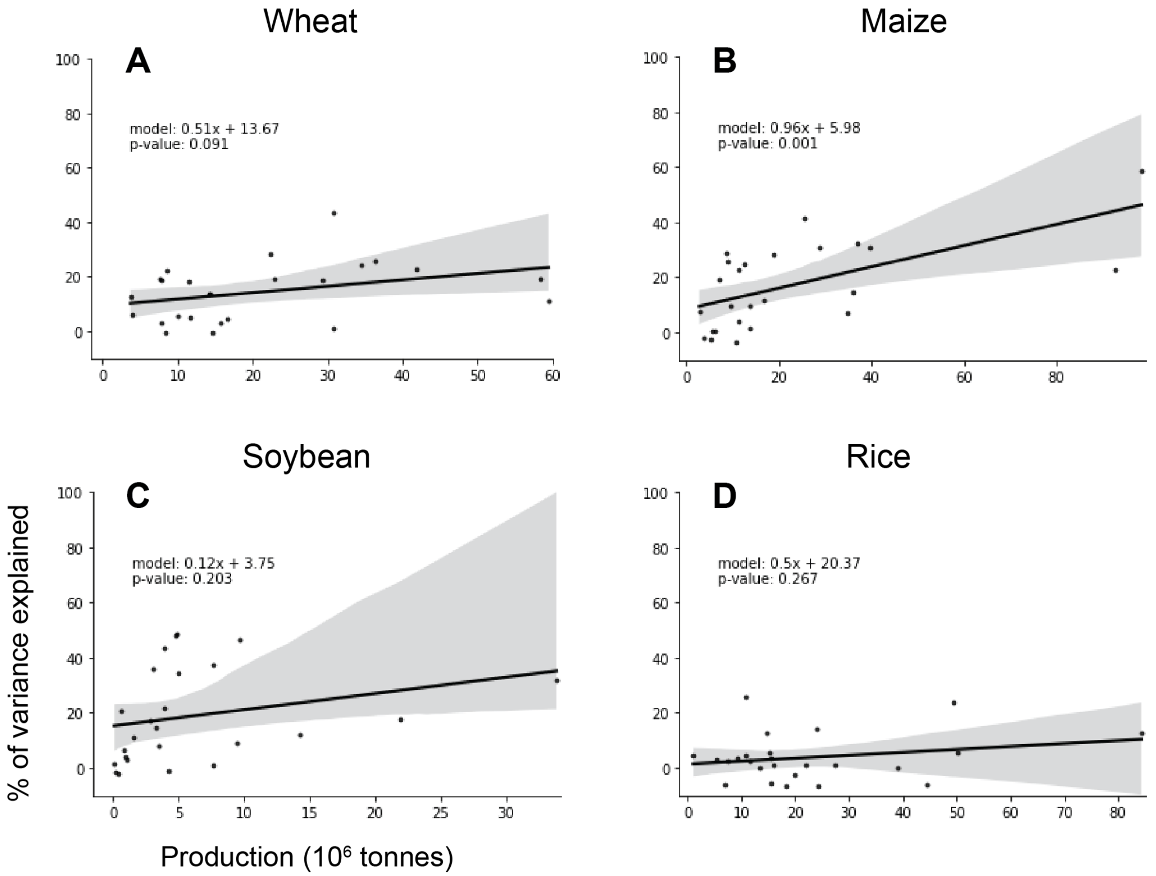

Fig. S4. The relationship between wheat (A), maize (B), soybean (C), and rice (D) production and proportion of their yield variance explained by climate variability. Each point represents one climate zone.

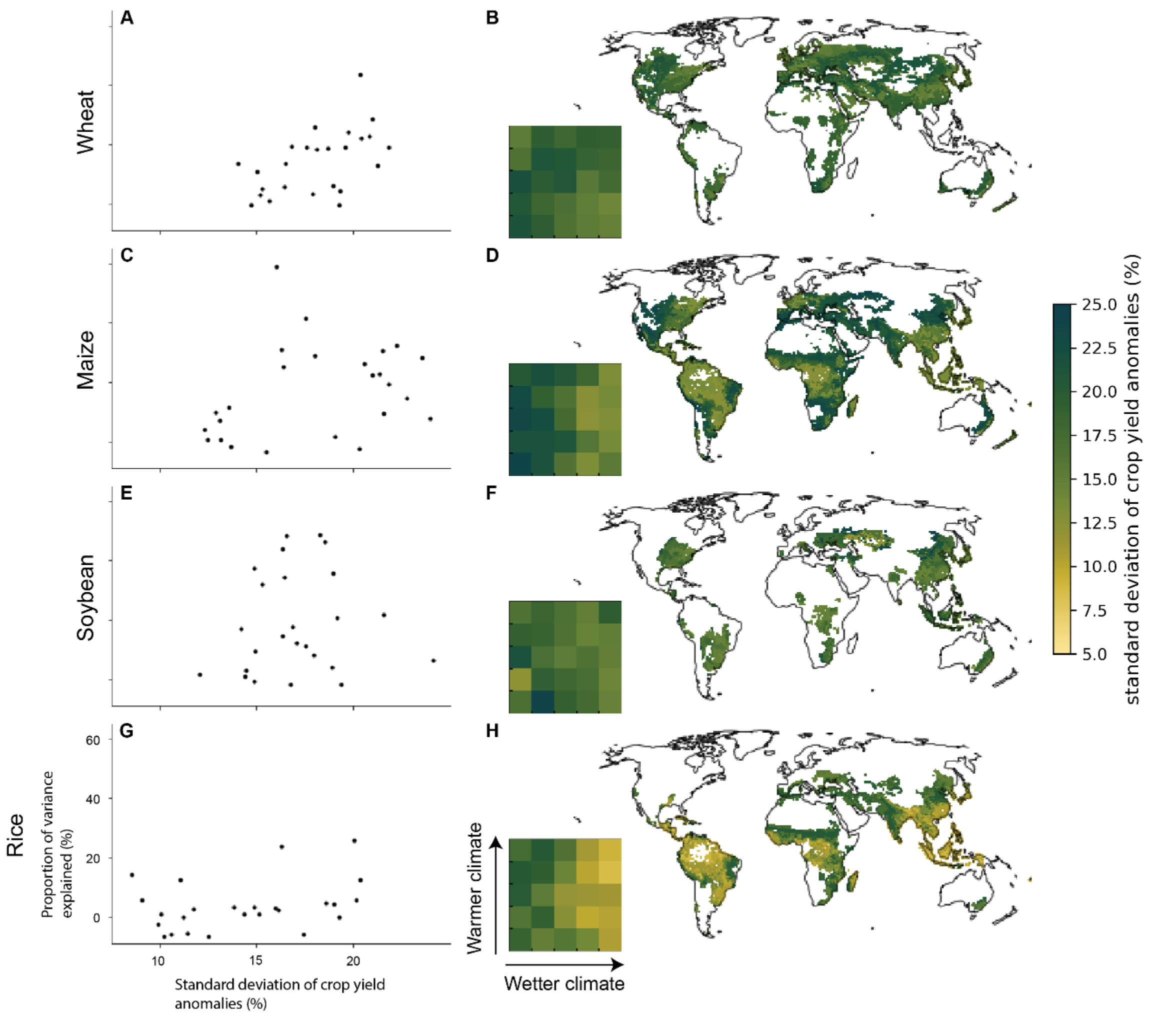

Fig. S5. The relationship between wheat (A, B), maize (C, D), soybean (E, F), and rice (G, H) yield anomaly variability and proportion of their yield anomaly variance explained by climate variations at climate zone level. In the scatter plot, each point represents one climate zone, whereas the mapped results show the standard deviation of crop yield anomalies for each climate bin.

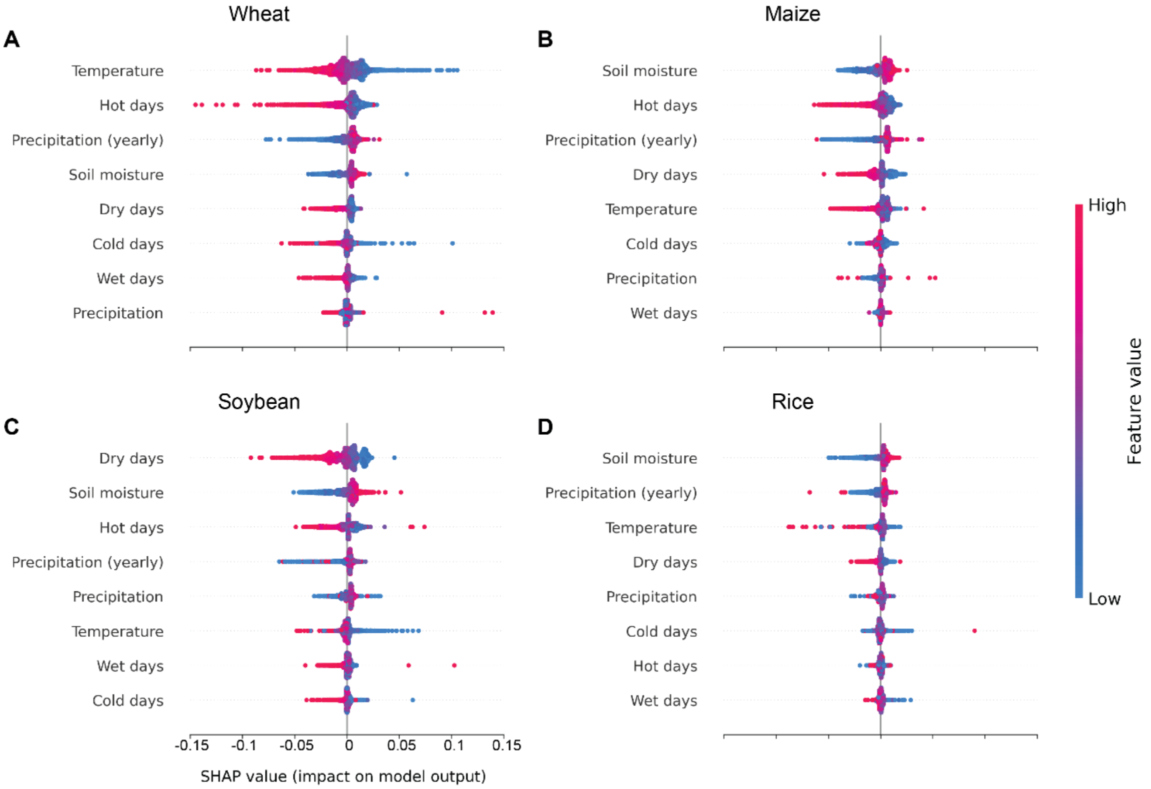

Fig. S6. SHAP summary plots for wheat (A), maize (B), soybean (C), and rice (D) derived from the globally fitted XGBoost regression models of the main analyses. Each point represents the Shapley value, i.e., the contribution of the climatological variable (feature) to the predicted outcome, for an instance of the data[9]. The climatological variables are ordered by their importance in the model output, the top variable being the most important.

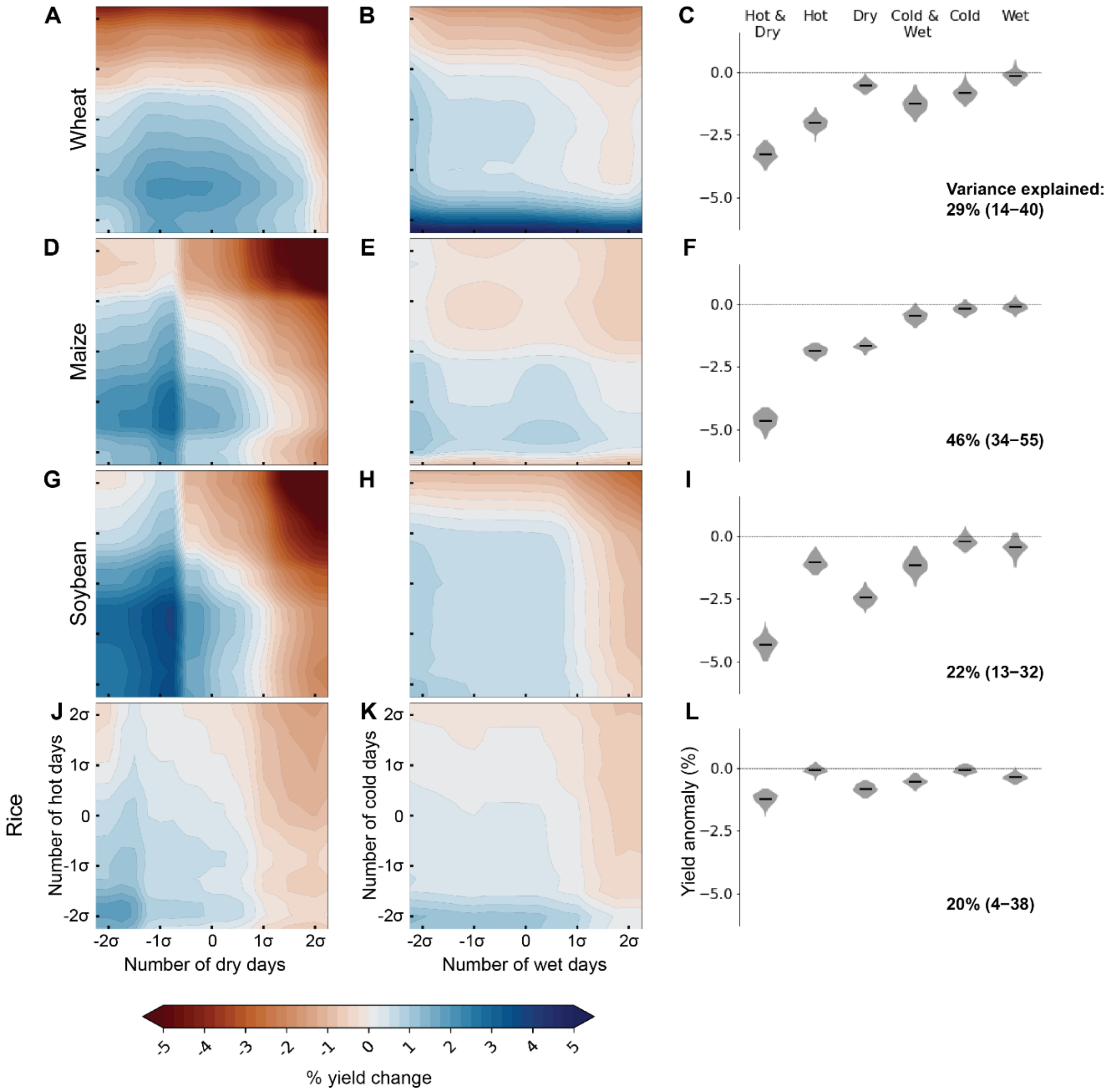

Fig. S7. Same as Fig. 2, but with GLEAM[3] soil moisture data.

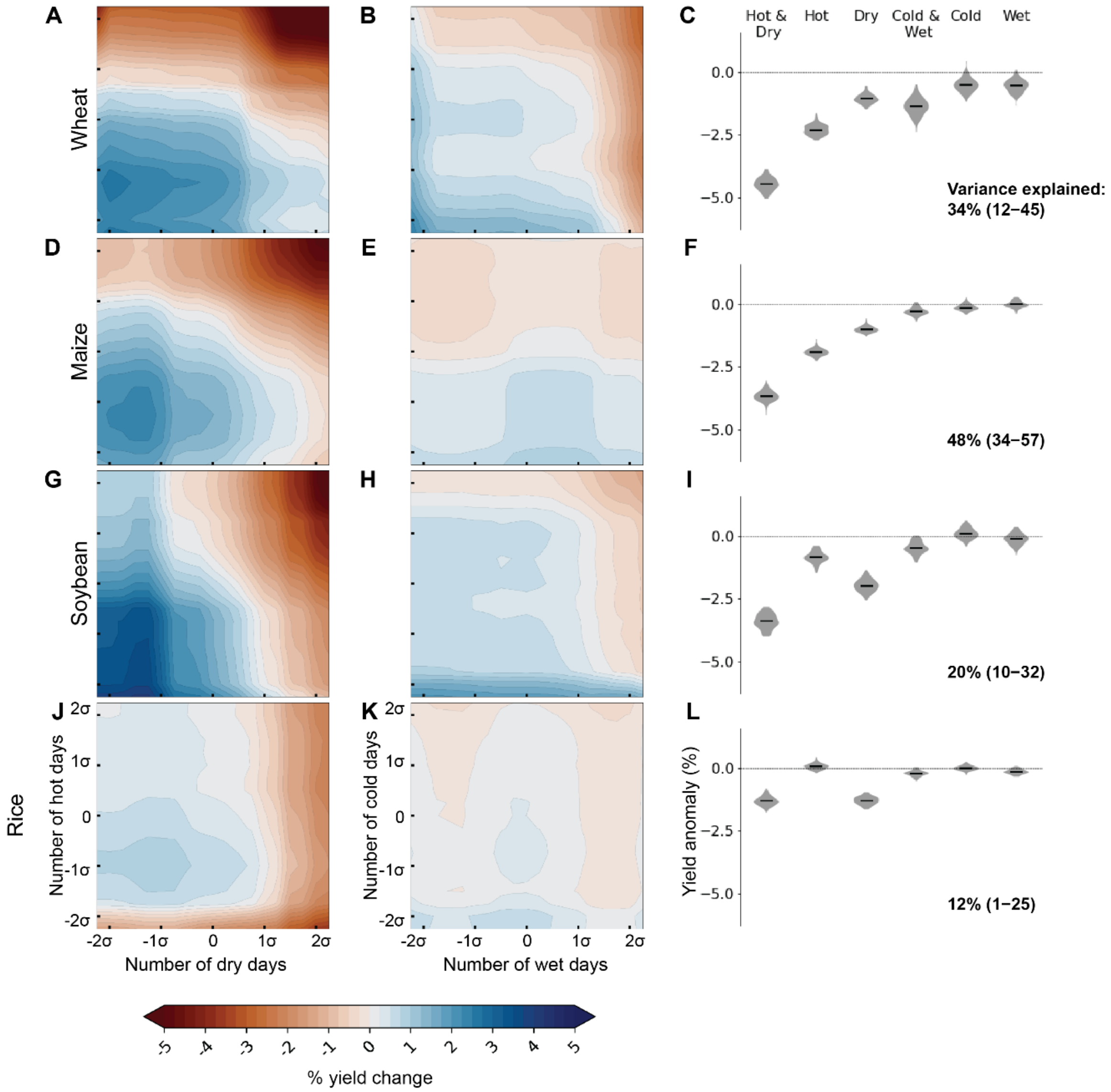

Fig. S8. Same as Fig. 2, but with extreme indicators (i.e., anomaly in hot, dry, cold, and wet days during the growing season) also linearly de-trended.

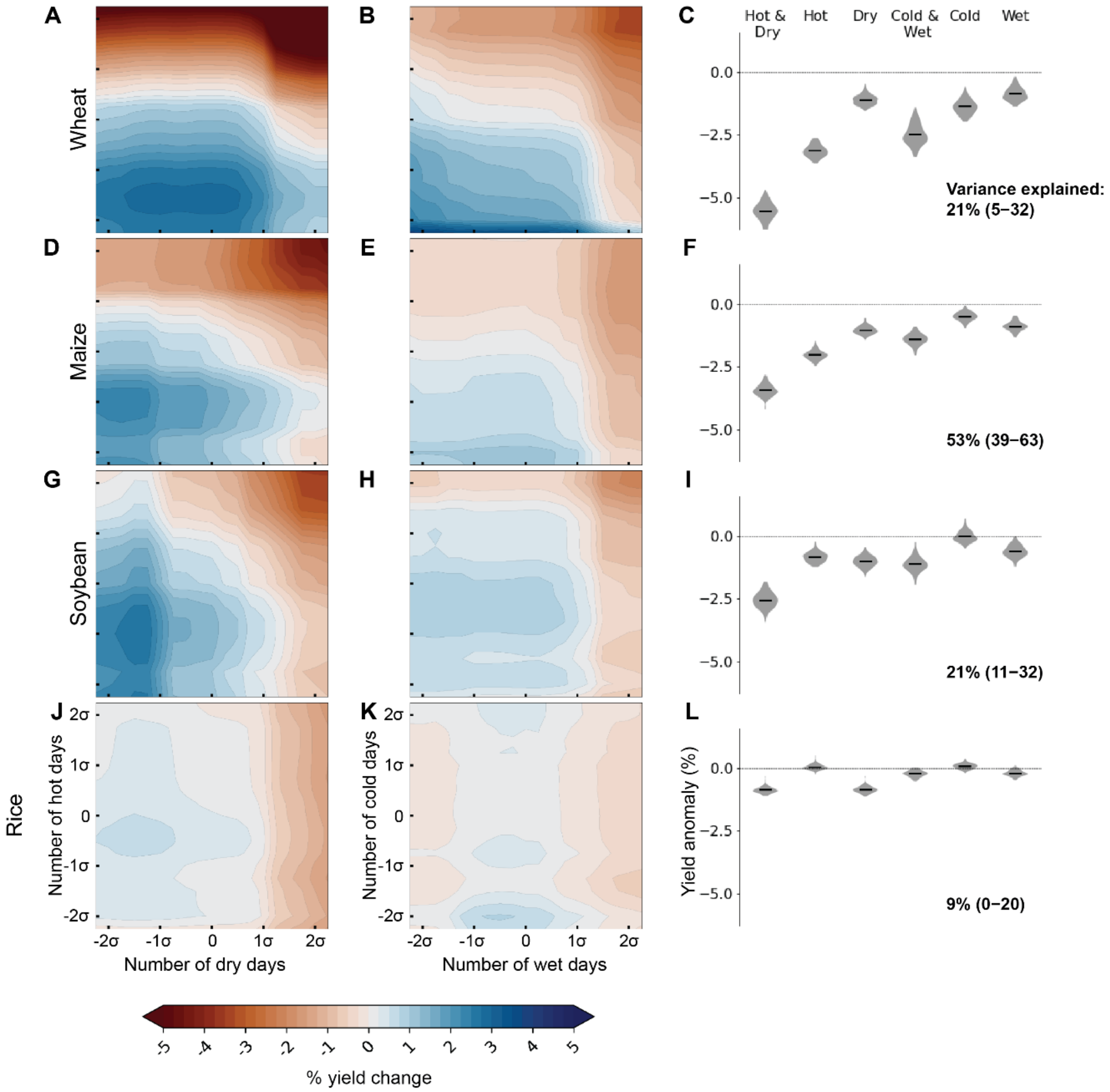

Fig. S9. Same as Fig. 2, but with full growing season[10] climate data.

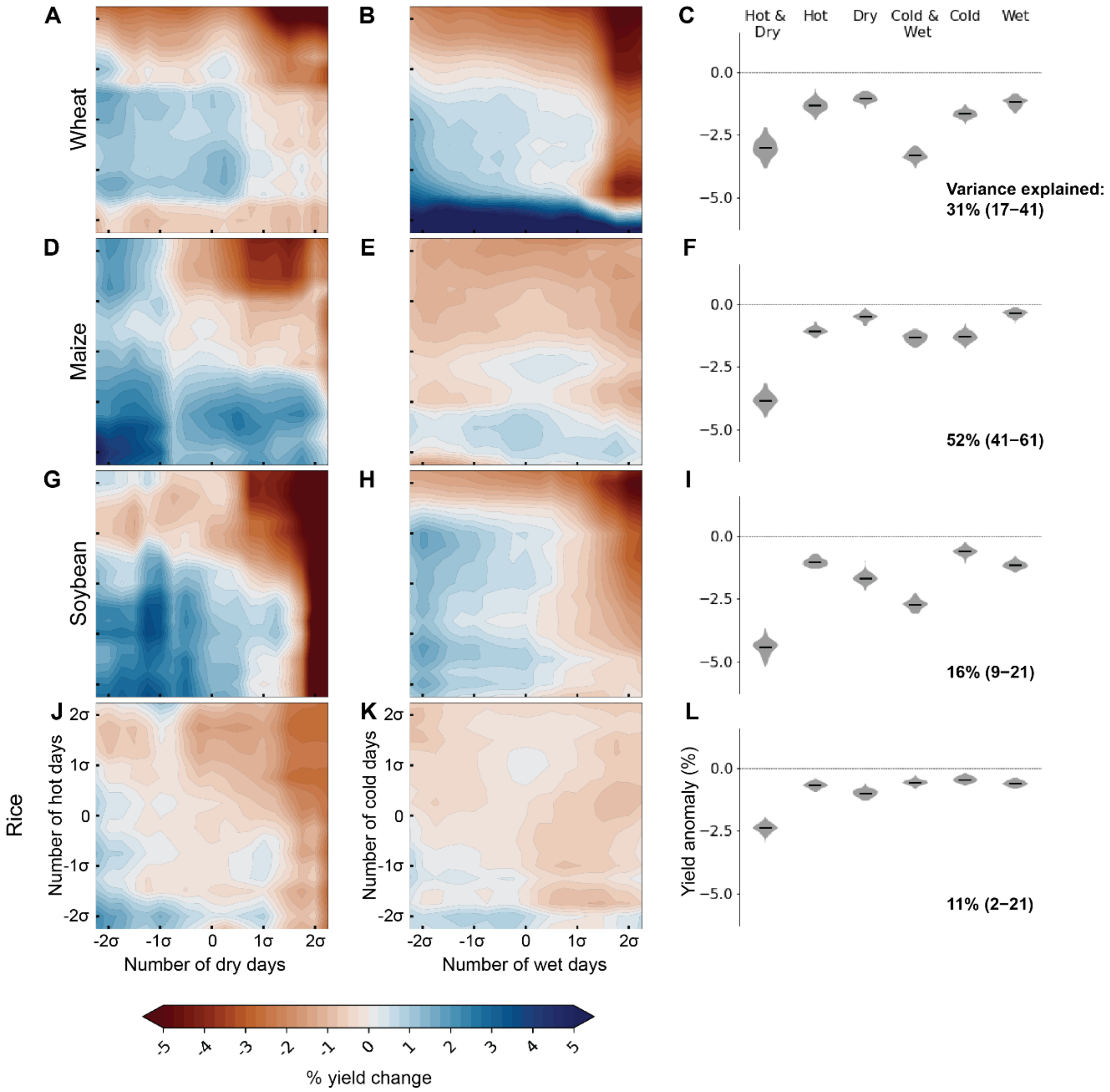

Fig. S10. Same as Fig. 2, but with a Random Forest model[7]. Here, Random Forest is trained with 50 trees so that in each tree, the nodes are expanded until all leaves are pure.

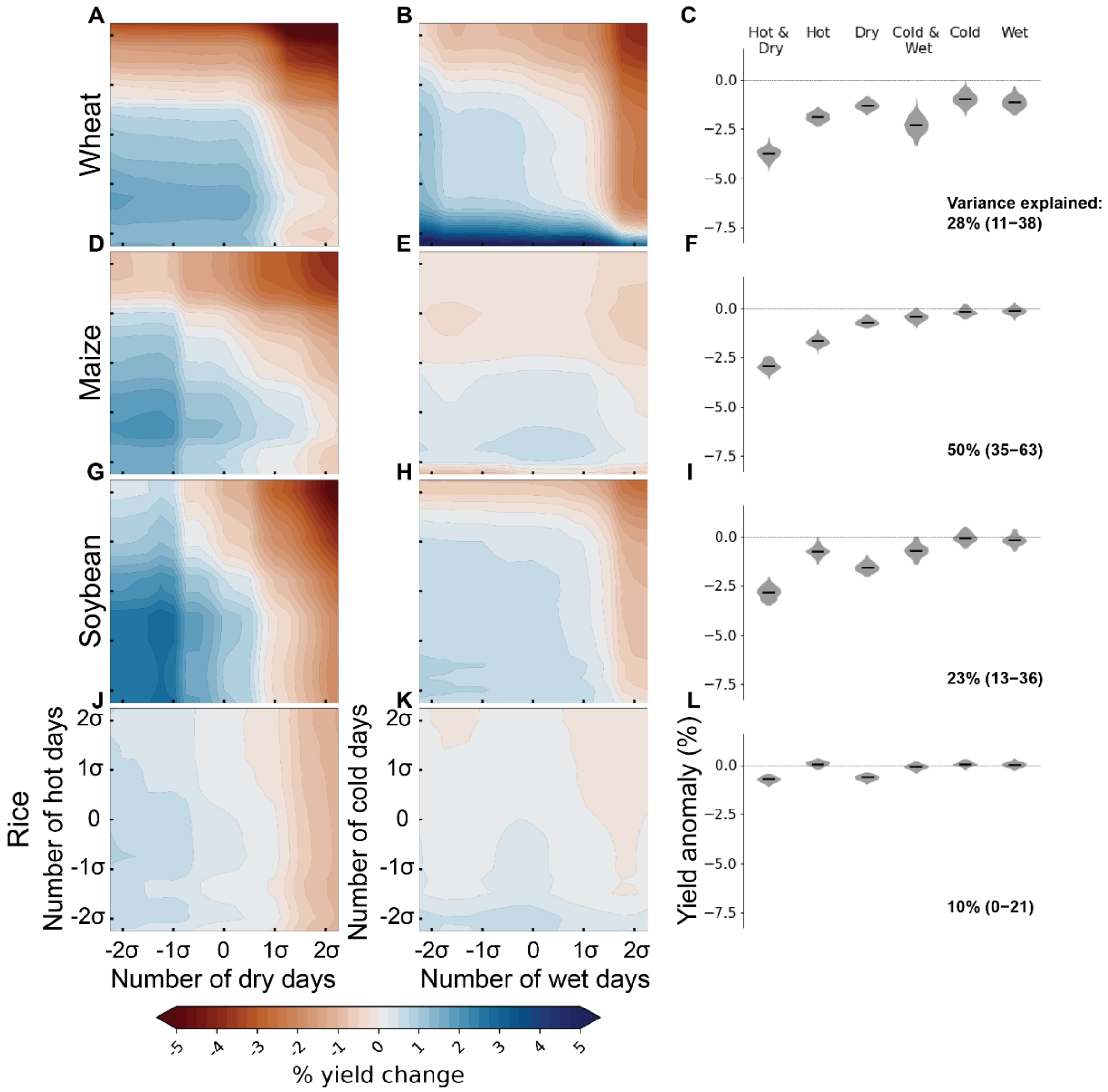

Fig. S11. Same as Fig. 2, but including also climatological mean growing season temperature and annual total precipitation, calculated across the study period, as explanatory variables.

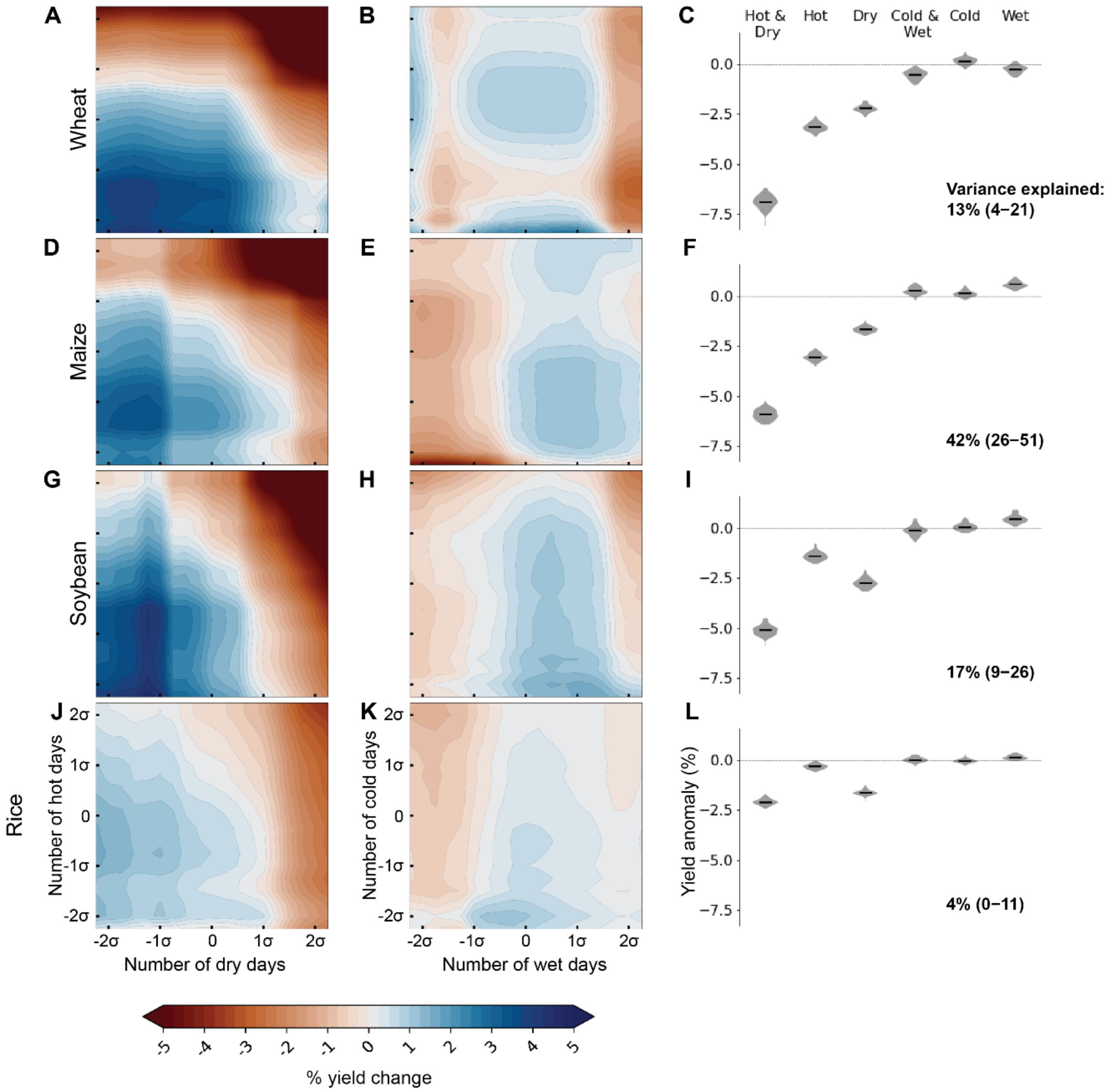

Fig. S12. Same as Fig. 2, but with solely the number of hot, dry, wet, and cold days as explanatory variables.

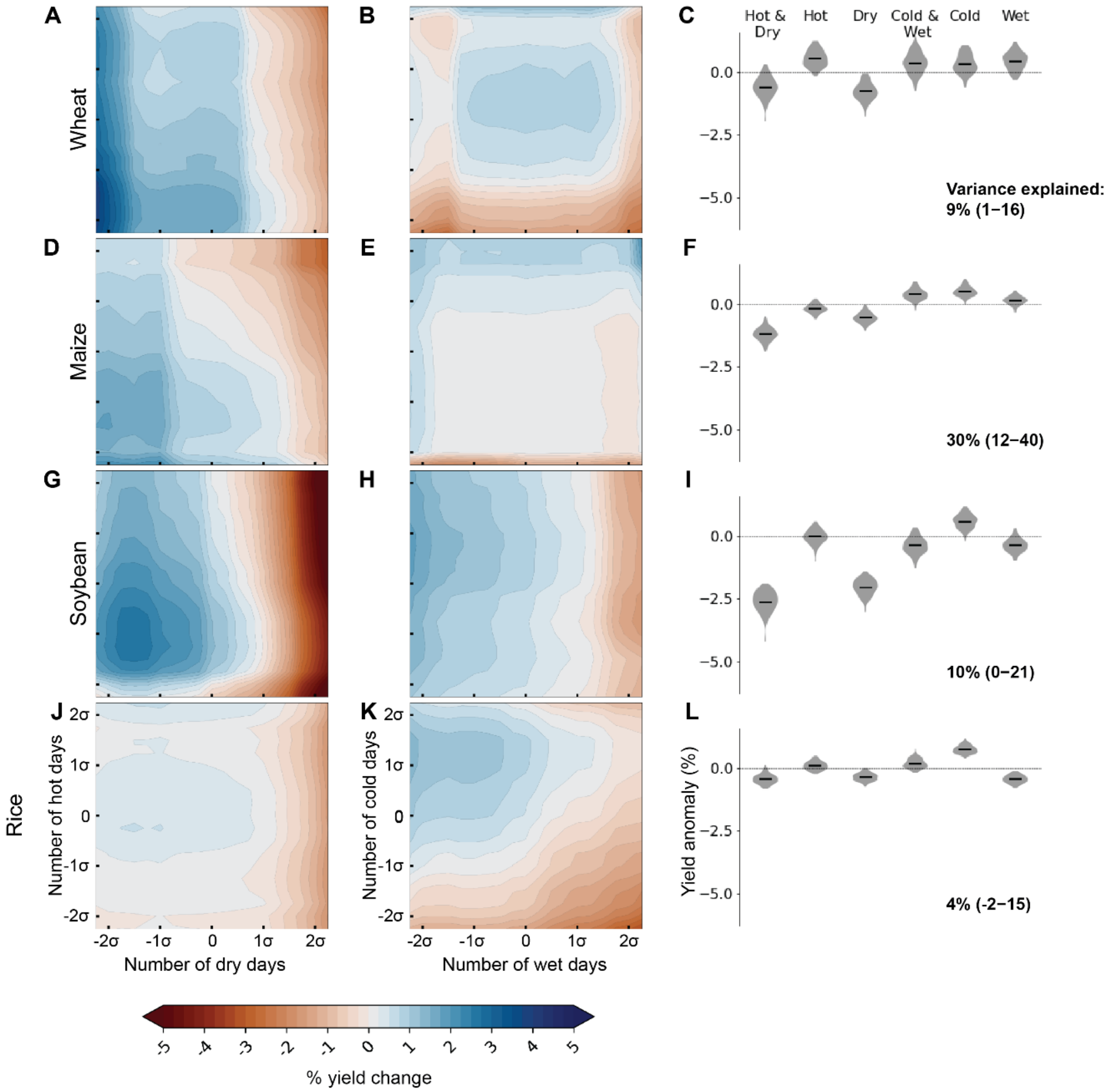

Fig S13. Same as Fig. 2, but with Iizumi and Sakai (2020)[5] crop yield data.

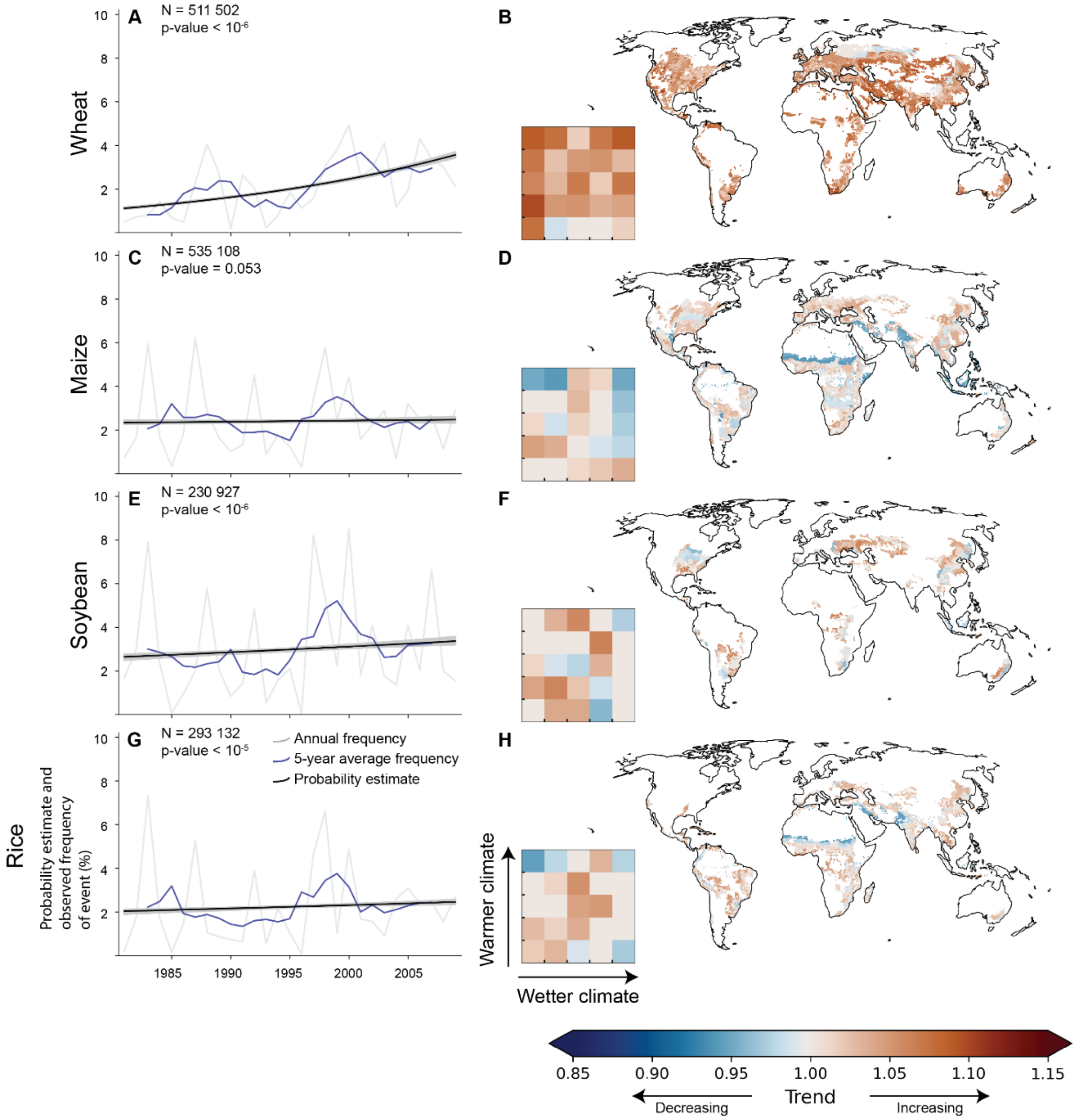

Fig. S14. Same as Fig. 4, but for GLEAM³ soil moisture data.

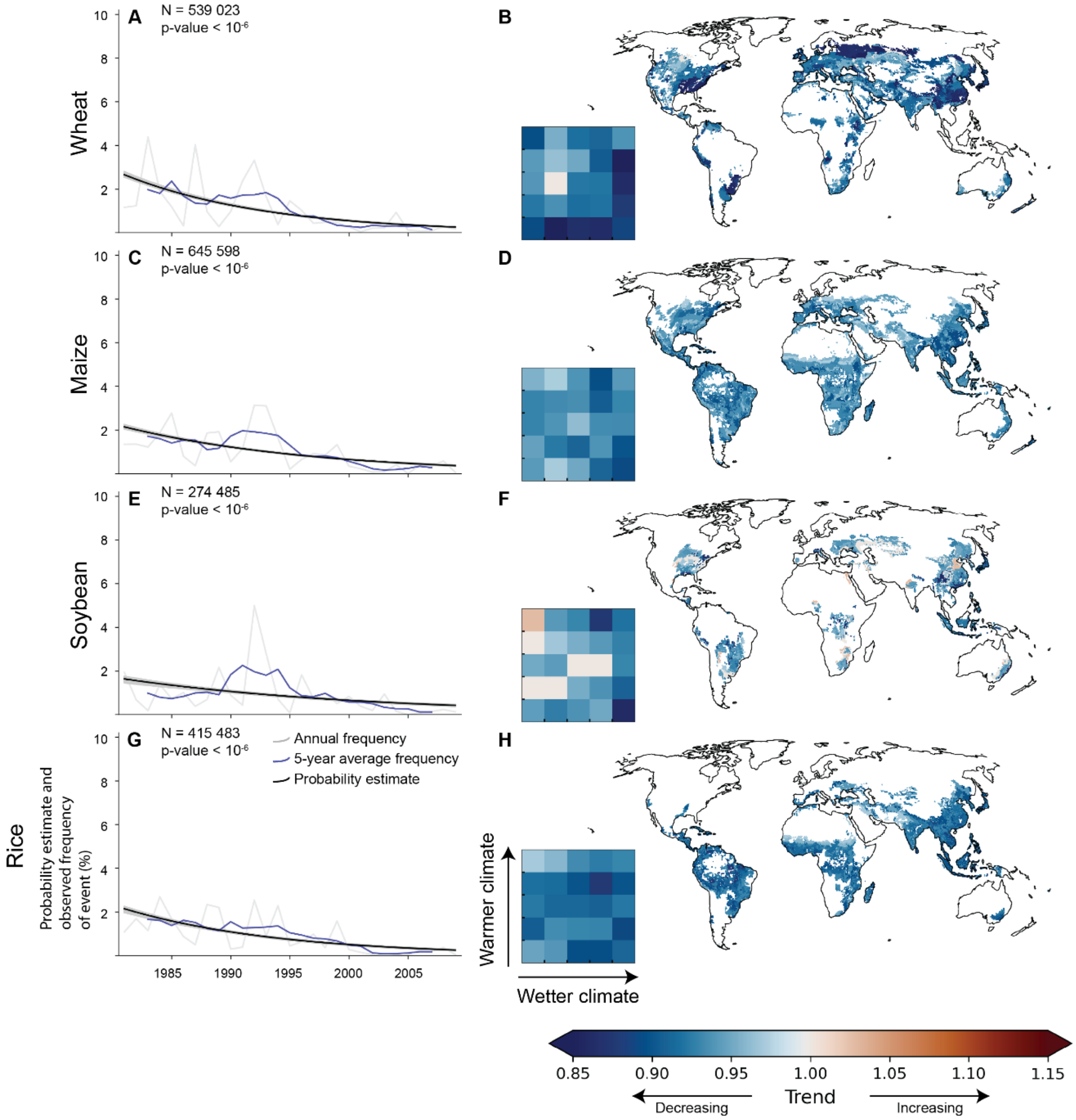

Fig. S15. Same as Fig. 4, but for wet and cold events.

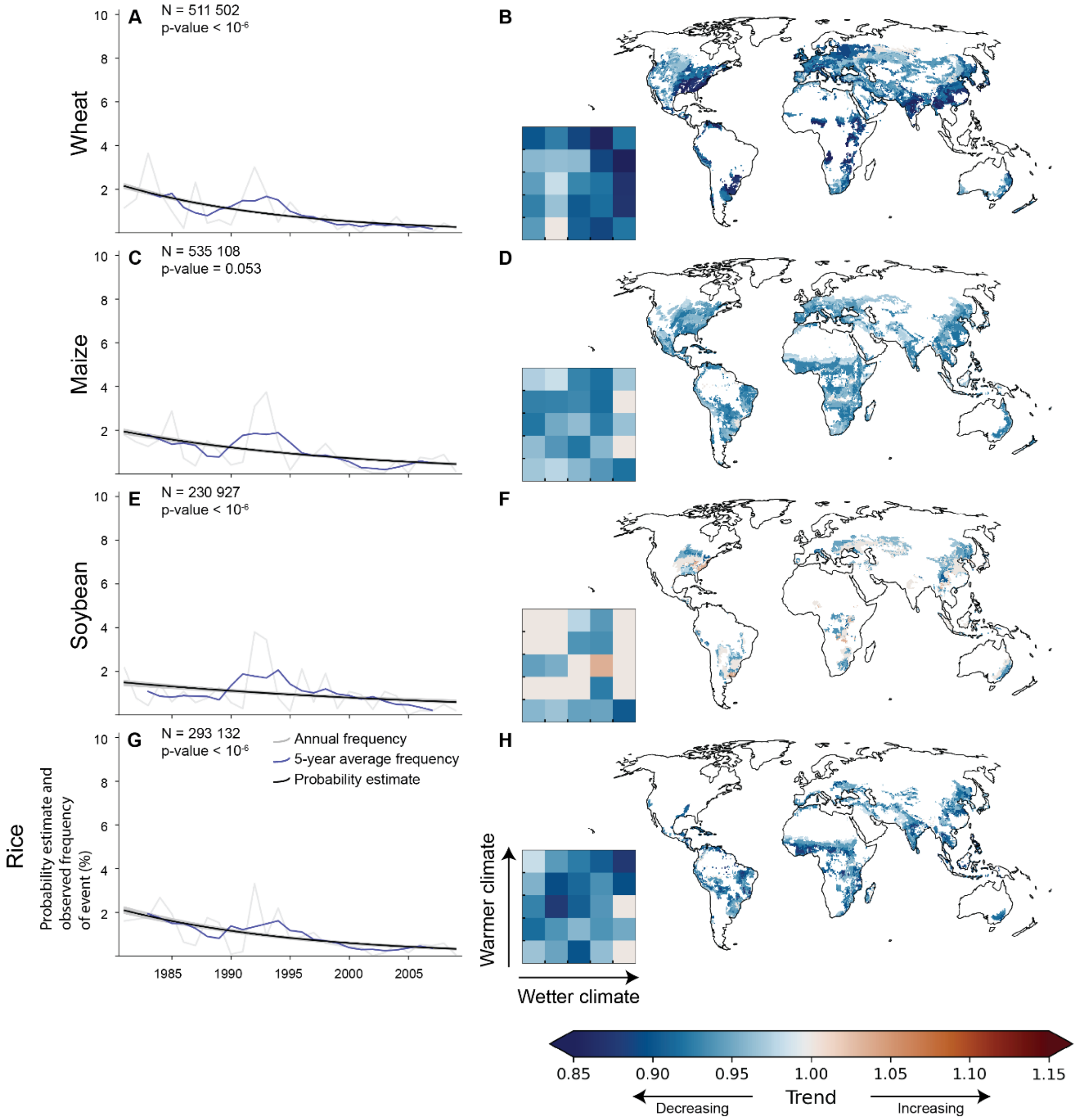

Fig. S16. Same as Fig. 4, but for wet and cold events and GLEAM[3] soil moisture data.

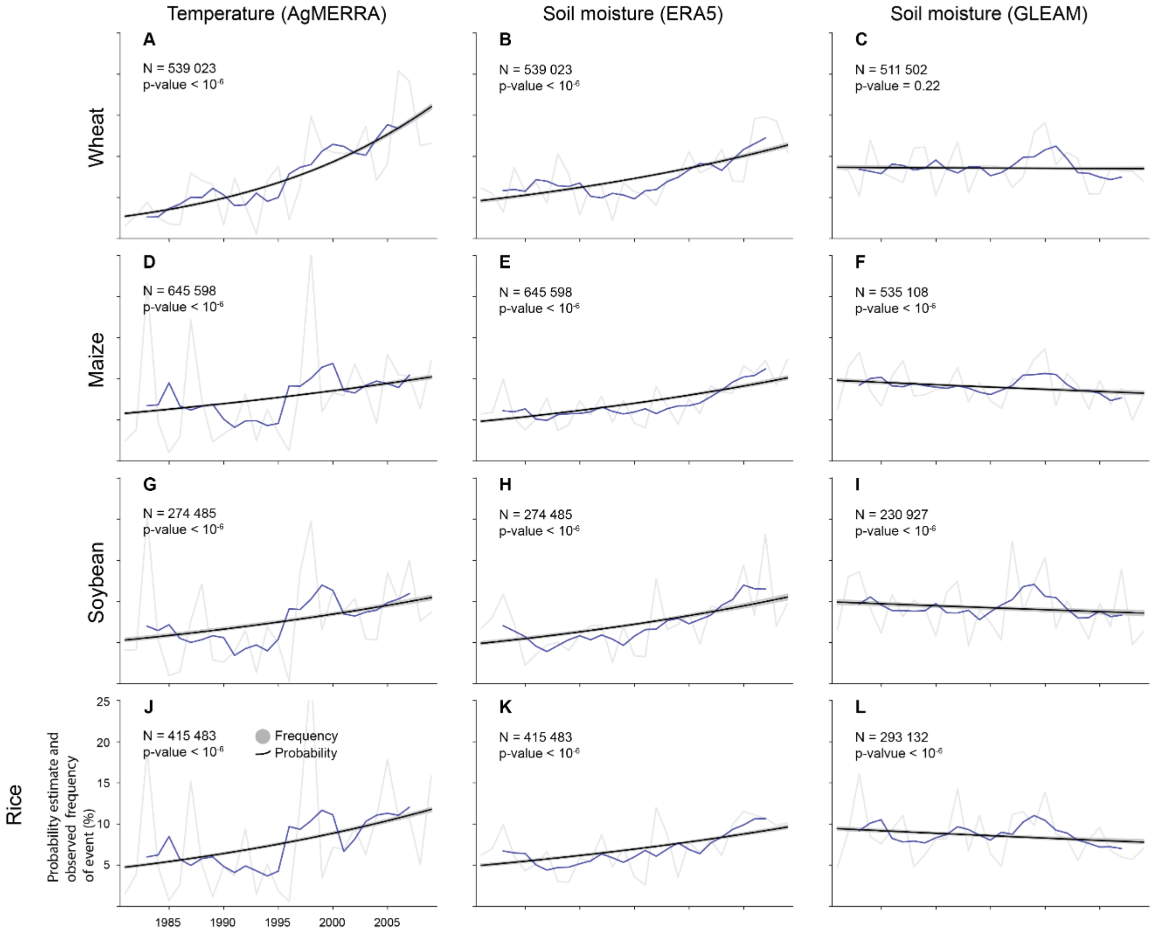

Fig. S17. Historical evolution in the probability of hot[1] (1) and dry[2,3] conditions between 1980–2009. Results are shown for studied crops: wheat (A,B,C), maize (D,E,F), soybean (G,H,I), and rice (J,K,L). The analysis was conducted utilizing data for all raster cells across the globe with both crop yield and weather data for the respective crop types. Here, a grid cell is considered hot (dry) for a specific year, if the number hot (dry) days during the growing season deviates at least 1.5σ from the long-term average. The historical evolution of the probability in hot (dry) events was assessed by logistic regression, while frequency was calculated as a percentage of hot (dry) events for each year and as a five-year average. The uncertainty intervals for the regression lines in the figure were calculated by bootstrapping (N=100) the observations and plotting the regression line for each sample (in gray color).

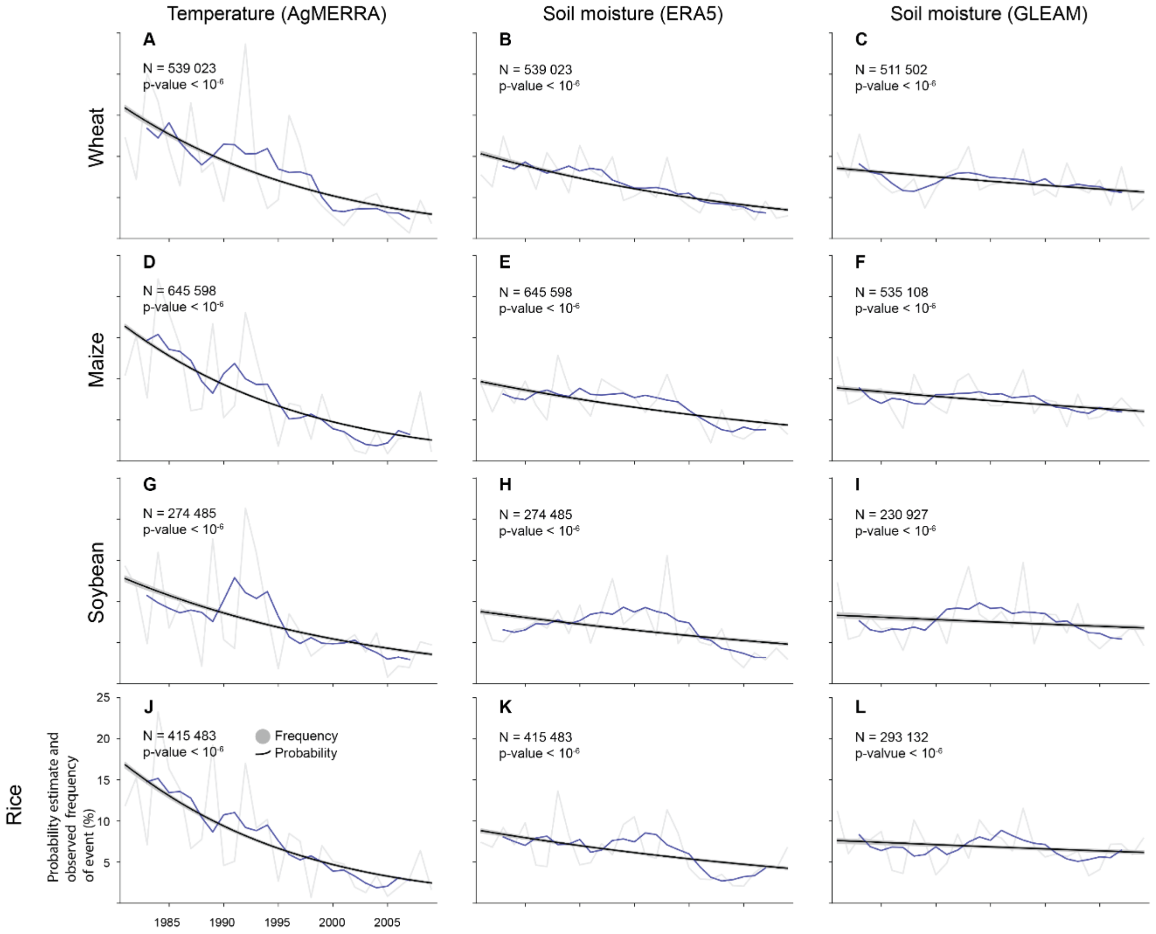

Fig. S18. Historical evolution in the probability of cold[1] and wet[2,3] conditions between 1980–2009. Results are shown for studied crops: wheat (A,B,C), maize (D,E,F), soybean (G,H,I), and rice (J,K,L). The analysis was conducted utilizing data for all raster cells across the globe with both crop yield and weather data for the respective crop types. Here, a grid cell is considered cold (wet) for a specific year, if the number cold (wet) days during the growing season deviates at least 1.5σ from the long-term average. The historical evolution of the probability in cold (wet) events was assessed by logistic regression, while frequency was calculated as a percentage of cold (wet) events for each year and as a five-year average. The uncertainty intervals for the regression lines in the figure were calculated by bootstrapping (N=100) the observations and plotting the regression line for each sample (in gray color).

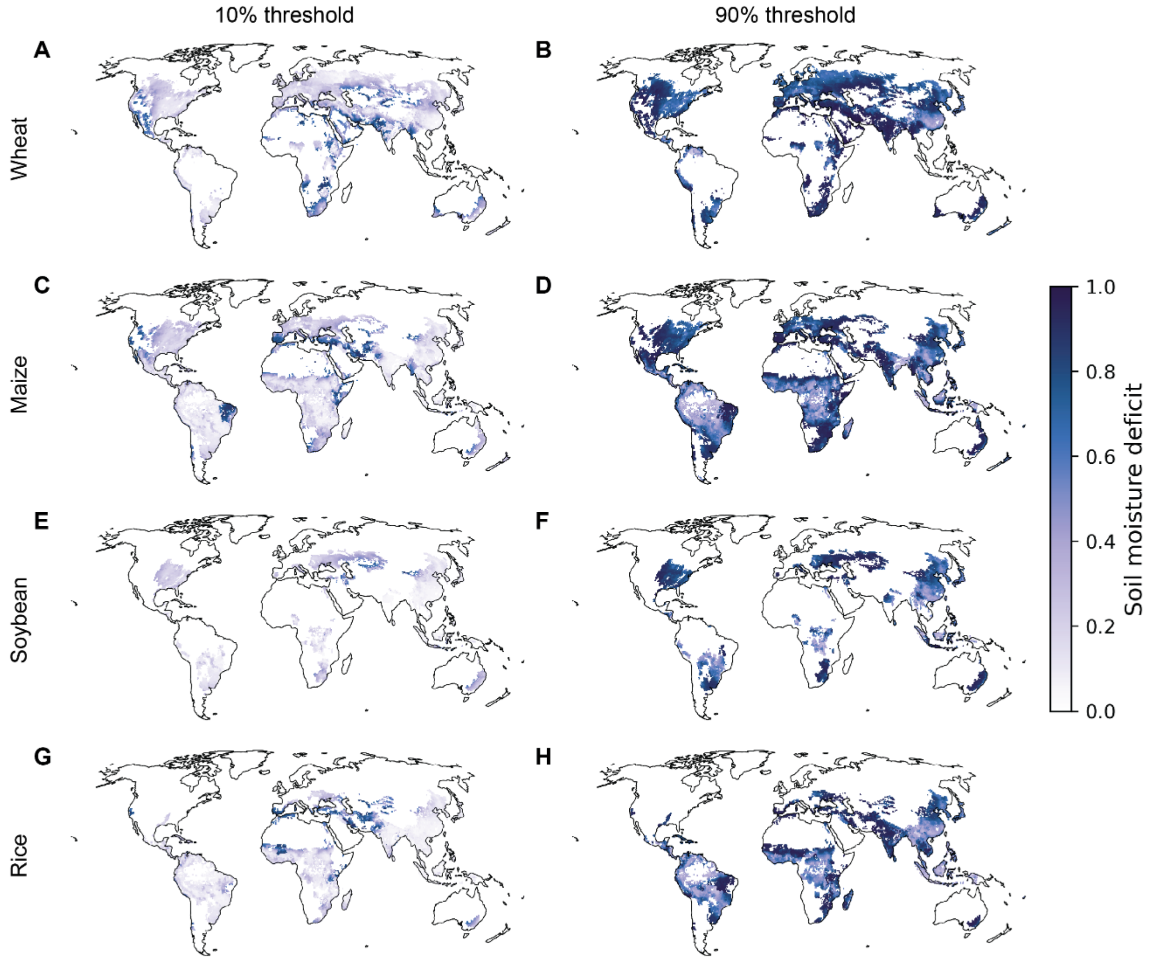

Fig. S19. 10% (A,C,E,G) and 90% (B,D,F,H) percentiles for soil moisture deficit (derived from ERA5[2]) for each raster cell during wheat (A,B), maize (C,D), soybean (E,F), and rice (G,H) growing seasons.

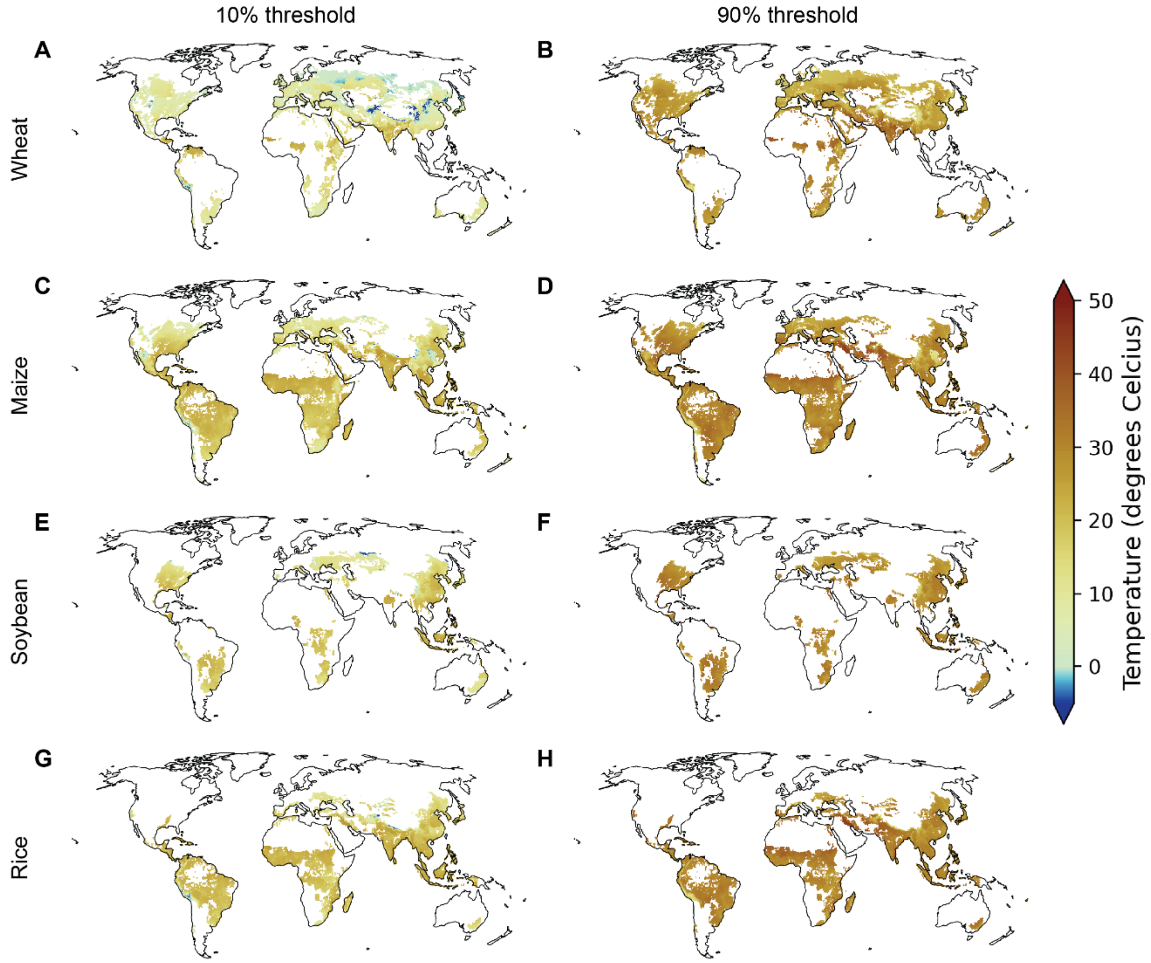

Fig. S20. 10% (A,C,E,G) and 90% (B,D,F,H) percentiles for temperature[1] for each raster cell during wheat (A,B), maize (C,D), soybean (E,F), and rice (G,H) growing seasons.

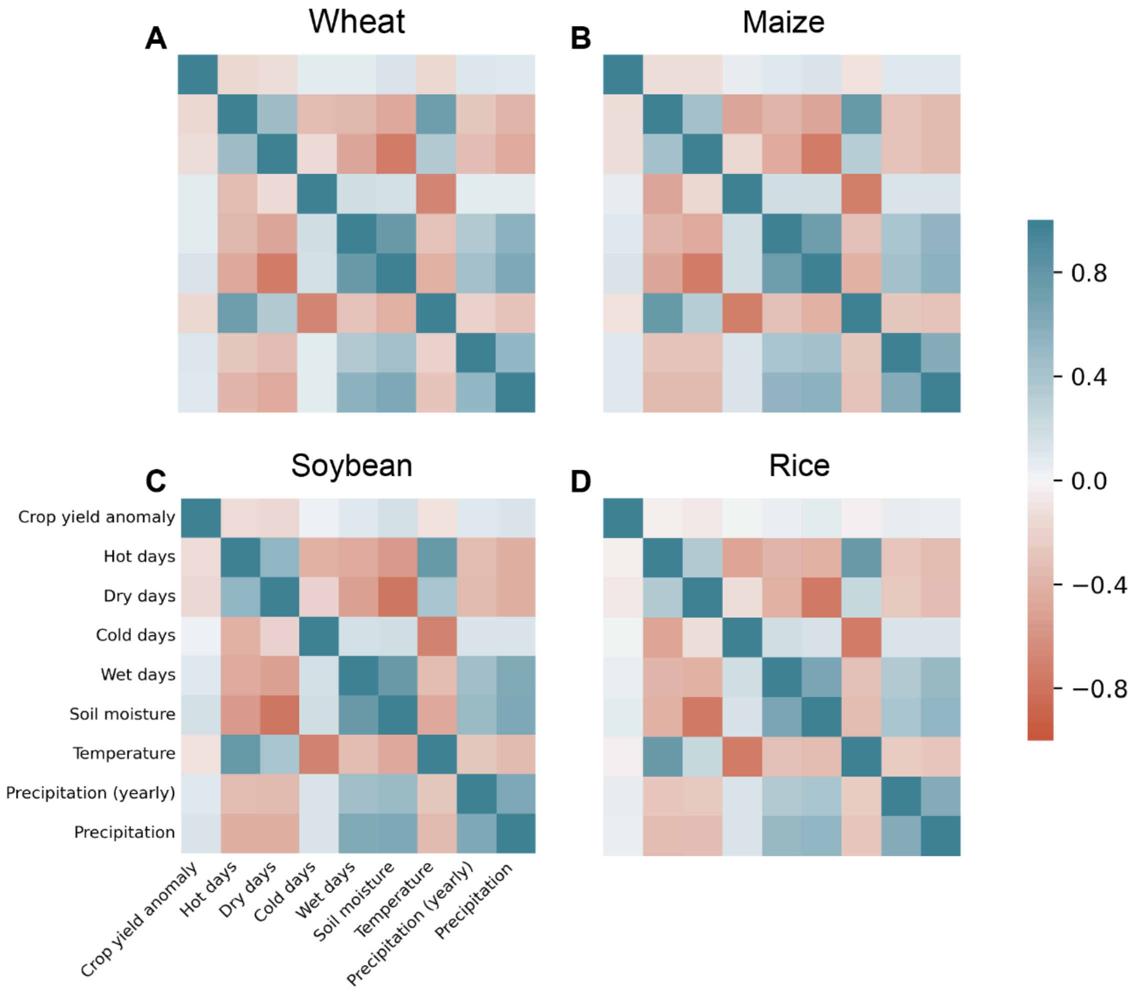

Fig. S21. Pearson's correlation matrices of yield anomalies and the growing season climate indicators for wheat (A), maize (B), soybean (C), and rice (D) across all grid cells included in the global regression analyses conducted with the Ray et al. (2019)[4] crop yield data and presented in the main text.

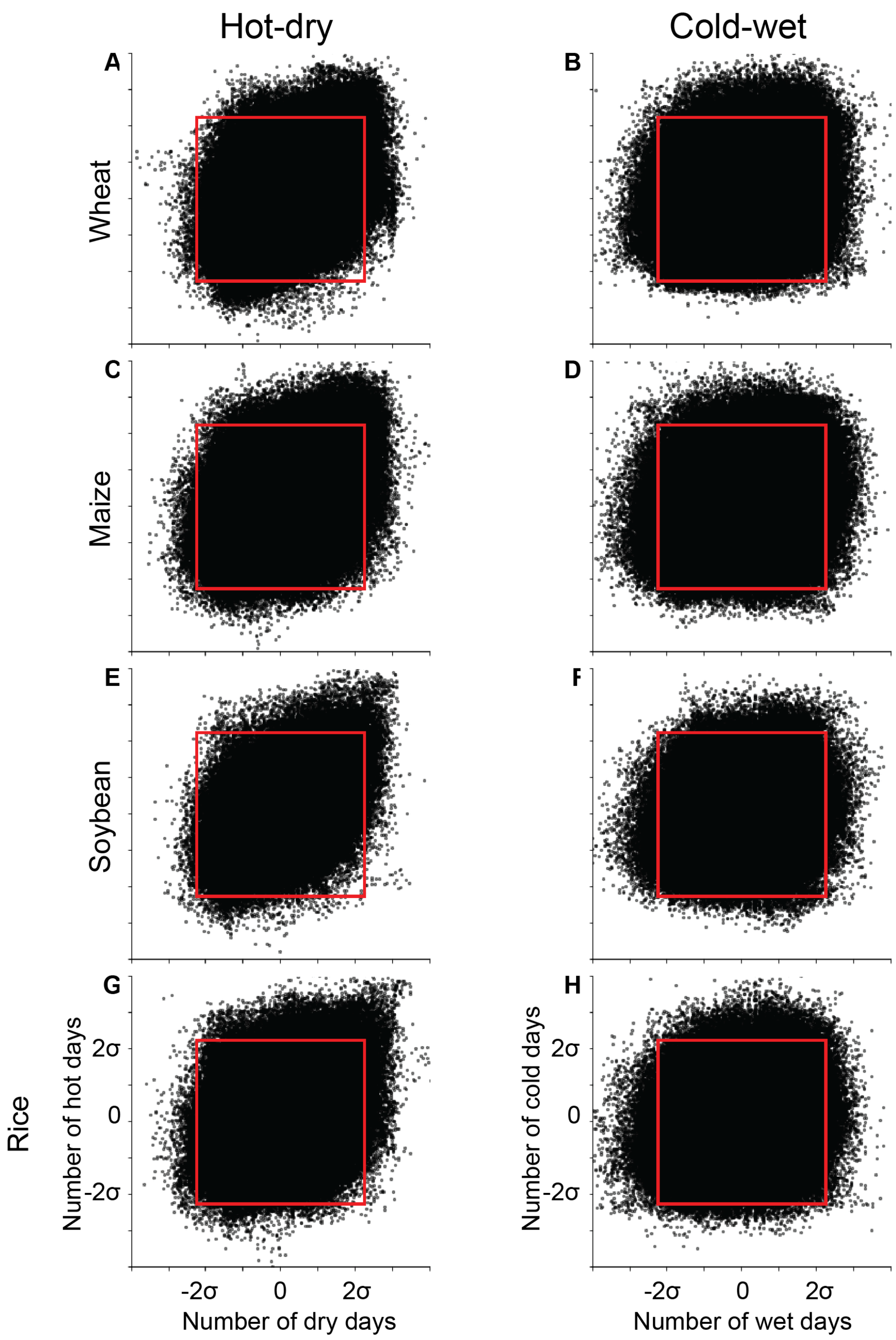

Fig. S22. Scatter plots showing the relationship between hot, and dry (A, C, E, G) as well as cold, and wet (B, D, F, H) conditions for the growing seasons of wheat (A, B), maize (C, D), soybean (E, F), and rice (G, H). Data are shown across all grid cells and years included in the global regression analyses conducted with the Ray et al. (2019) crop yield data[4]. The red box refers to the extent of the data visualized in the partial dependence plots of Fig. 2.

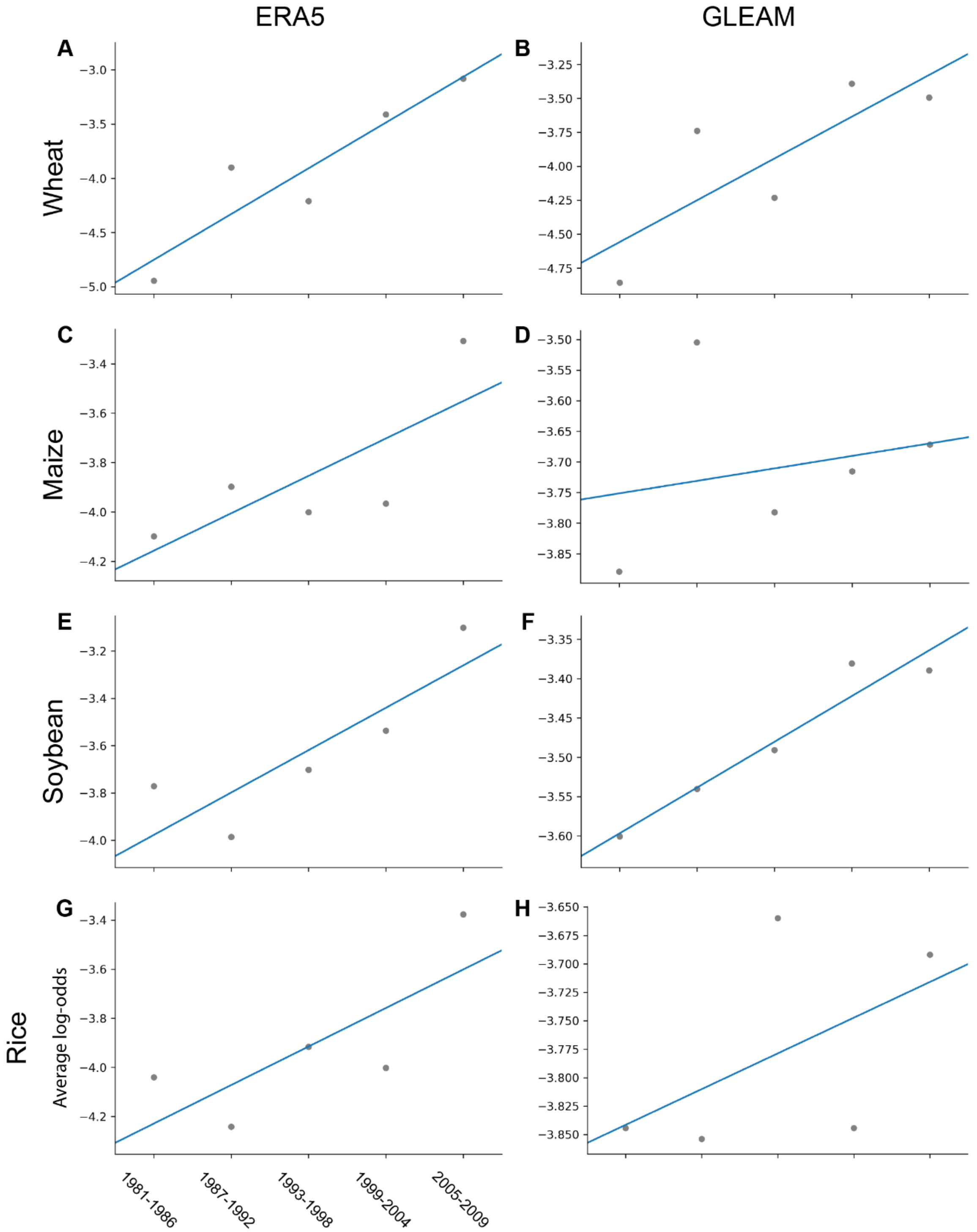

Fig. S23. Average log-odds values of co-occurring dry and hot events for each temporally defined quintile with ERA5[2] and GLEAM[3] soil moisture data. Results are shown for studied crops: wheat (A,B), maize (C,D), soybean (E,F), and rice (G,H).

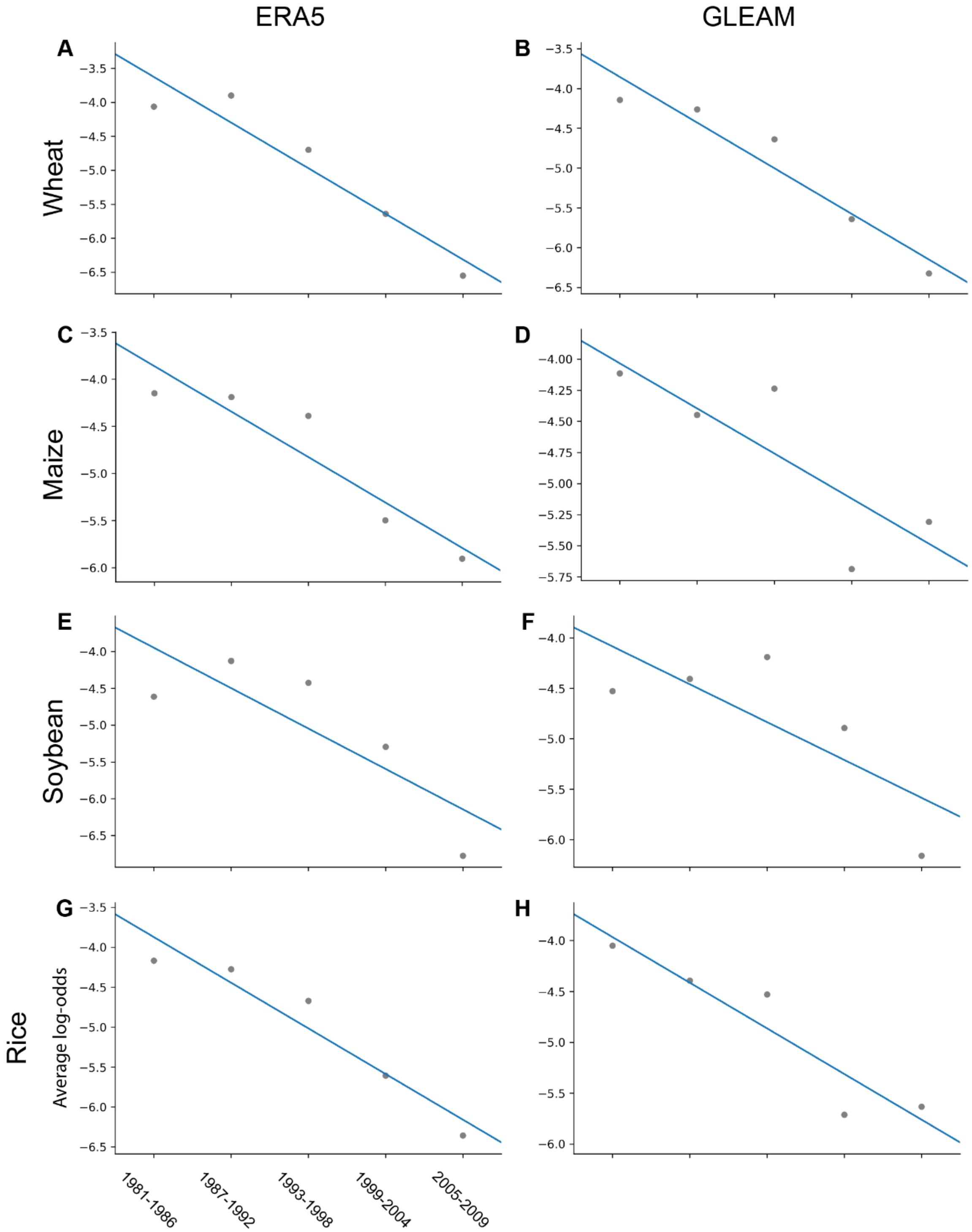

Fig. S24. Average log-odds values of co-occurring cold and wet events for each temporally defined quintile with ERA5[2] and GLEAM[3] soil moisture data. Results are shown for studied crops: wheat (A,B), maize (C,D), soybean (E,F), and rice (G,H).

SI References

1. Ruane, A. C., Goldberg, R. & Chryssanthacopoulos, J. Climate forcing datasets for agricultural modeling: Merged products for gap-filling and historical climate series estimation. *Agric.For.Meterol.* **200**, 233–248 (2015).

2. Hersbach, H. *et al.* The ERA5 global reanalysis. *Q.J.R.Meteorol.Soc.* (2020) doi:10.1002/qj.3803.

3. Martens, B. *et al.* GLEAM v3: Satellite-based land evaporation and root-zone soil moisture. *Geoscientific Model Dev.* **10**, 1903–1925 (2017).

4. Ray, D. K. *et al.* Climate change has likely already affected global food production. *PLoS ONE* **14**, (2019).

5. Iizumi, T. & Sakai, T. The global dataset of historical yields for major crops 1981–2016. *Sci.Data* **7**, (2020).

6. Chen, T. & Guestrin, C. Xgboost: A scalable tree boosting system. in 785–794 (2016).

7. Breiman, L. Random forests. *Mach Learn* **45**, 5–32 (2001).

8. Portmann, F. T., Siebert, S. & Döll, P. MIRCA2000—Global monthly irrigated and rainfed crop areas around the year 2000: A new high-resolution data set for agricultural and hydrological modeling. *Global Biogeochem.Cycles* **24**, (2010).

9. Lundberg, S. M. *et al.* From local explanations to global understanding with explainable AI for trees. *Nature machine intelligence* **2**, 56–67 (2020).

10. Müller, C. *et al.* Global gridded crop model evaluation: Benchmarking, skills, deficiencies and implications. *Geoscientific Model Dev.* **10**, 1403–1422 (2017).
